# Tetraspanin7 in adipose tissue remodeling and its impact on metabolic health

**DOI:** 10.1016/j.molmet.2025.102168

**Published:** 2025-05-12

**Authors:** Shino Nemoto, Kazuyo Uchida, Tetsuya Kubota, Manabu Nakayama, Yong-Woon Han, Shigeo Koyasu, Hiroshi Ohno

**Affiliations:** 1 Laboratory for Intestinal Ecosystem, RIKEN Center for Integrative Medical Sciences, Yokohama, Kanagawa, Japan; 2 Division of Diabetes and Metabolism, The Institute of Medical Science, Asahi Life Foundation, Tokyo, Japan; 3 Division of Cardiovascular Medicine, Toho University Ohashi Medical Center, Tokyo, Japan; 4 Department of Diabetes and Metabolic Diseases, Graduate School of Medicine, The University of Tokyo, Tokyo, Japan; 5 Laboratory of Medical Omics Research, Department of Frontier Research and Development, Kazusa DNA Research Institute, Kisarazu, Chiba, Japan; 6 Laboratory for Integrative Genomics, RIKEN Center for Integrative Medical Sciences, Yokohama, Kanagawa, Japan; 7 Laboratory for Immune Cell Systems, RIKEN Center for Integrative Medical Sciences, Yokohama, Kanagawa, Japan; 8 Laboratory for Immune Regulation, Graduate School of Medical and Pharmaceutical Sciences, Chiba University, Chiba, Chiba, Japan

**Keywords:** Tetraspanin7, Obesity, Fat distribution, Fat remodeling, Lipid droplet, Insulin resistance, Visceral-to-subcutaneous fat ratio

## Abstract

**Objective:**

We previously identified tetraspanin 7 (*Tspan7*) as a candidate gene influencing body weight in an obesity-related gene screening study. However, the mechanisms underlying its involvement in body weight regulation remained unclear. This study aims to investigate the role of TSPAN7 from a metabolic perspective.

**Methods:**

We utilized genetically modified mice, including adipose tissue-specific *Tspan7*-knockout and *Tspan7*-overexpressing models, as well as human adipose-derived stem cells with TSPAN7 knockdown and overexpression. Morphological, molecular, and omics analyses, including proteomics and transcriptomics, were performed to investigate TSPAN7 function. Physiological effects were assessed by measuring blood markers associated with lipid regulation under metabolic challenges, such as high-fat feeding and aging.

**Results:**

We show that TSPAN7 is involved in regulating lipid droplet formation and stabilization. *Tspan7*-knockout mice exhibited an increased proportion of small-sized adipocytes and a reduced visceral-to-subcutaneous fat ratio. This shift in fat distribution was associated with improved insulin sensitivity and altered branched-chain amino acid metabolism, as evidenced by increased expression of the branched-chain α-keto acid dehydrogenase complex subunit B in *Tspan7*-modified mice. Mechanistically, TSPAN7 deficiency promoted subcutaneous fat expansion, alleviating metabolic stress on visceral fat, a major contributor to insulin resistance.

**Conclusions:**

TSPAN7 influences lipid metabolism by modulating adipose tissue remodeling, particularly under metabolic challenges, such as high-fat diet exposure and aging. Its modulation enhances subcutaneous fat storage capacity while mitigating visceral fat accumulation, leading to improved insulin sensitivity. These findings position TSPAN7 as a potential target for therapeutic interventions aimed at improving metabolic health and preventing obesity-related diseases.

## Highlights

• TSPAN7 regulates lipid droplet formation and stabilization in adipocytes.
• TSPAN7 knockout increases small adipocytes and improves insulin sensitivity.
• Reduced visceral-to-subcutaneous fat ratio linked to TSPAN7 deletion in mice.
• Altered branched-chain amino acid metabolism observed in TSPAN7-modified mice.
• TSPAN7 modulation offers potential for obesity and metabolic disease therapies.

## 1 Introduction

Tetraspanin7 (*TSPAN7*), a member of the tetraspanin family, encodes a membrane protein involved in cell–cell interactions and signal transduction [1,2]. TSPAN7 is also known as the T-cell acute lymphoblastic leukemia antigen (TALLA) owing to its high expression in patients with T-cell acute lymphoblastic leukemia (T-ALL) and acute myeloid leukemia [3,4]. Several studies have investigated the role of TSPAN7 in immune cells, blood cells, and cancer cells [[5], [6], [7], [8]]. TSPAN7 is also highly expressed in the brain, which is indicative of its potential involvement in neurological diseases [[9], [10], [11], [12]]. Furthermore, recent studies have identified TSPAN7 as a potential autoantigen in type 1 diabetes [[13], [14], [15]], highlighting its involvement in immune-related metabolic regulation. Considering its involvement in diverse biological processes, TSPAN7 could also play an as yet unrecognized role in metabolic regulation.

In a previous study, we identified *Tspan7* as a candidate gene in subcutaneous adipose tissue through obesity-related gene screening [16]. Subsequent analyses, including Gene Expression Omnibus data mining and in-house validation experiments, consistently demonstrated an inverse correlation between TSPAN7 expression and body weight [16]. To investigate its functional relevance, we conducted experiments using adipose tissue-specific *Tspan7*-knockout and -overexpressing mouse models. These experiments demonstrated that *Tspan7* influences body weight regulation, prompting us to explore the underlying mechanisms. Given that the tetraspanin family plays essential roles in cellular organization and signaling [17,18], we hypothesized that TSPAN7 might regulate adipocyte function, lipid storage, and fat distribution, thereby influencing metabolic homeostasis and obesity. However, the specific role of TSPAN7 in adipose tissue biology remains unexplored.

Body weight regulation is governed by complex genetic (e.g., sex and genomic variations) and environmental (e.g., diet and physical activity) factors as well as age. Among these factors, fat distribution, particularly the balance between subcutaneous and visceral fat, plays a significant role in metabolic health. Visceral fat is metabolically more active than subcutaneous fat and releases higher levels of free fatty acids (FFAs), which may disrupt energy balance and promote weight gain [[19], [20], [21]]. Genetic studies have suggested that visceral fat accumulation may be causally linked to the development of metabolic disorders, such as insulin resistance and type 2 diabetes [22,23]. Visceral fat is widely recognized as a risk factor for obesity-related health issues, including chronic inflammation, metabolic syndrome, hypertension, and cardiovascular diseases [24], whereas subcutaneous fat has been proposed to play a protective role by buffering lipid overflow and maintaining metabolic homeostasis [[25], [26], [27], [28]]. Despite these insights, the molecular mechanisms governing fat distribution remain poorly understood.

In this study, we investigated the role of TSPAN7 in body weight regulation by examining its effects on fat distribution and metabolic health. In particular, we aimed to extend our previous findings by elucidating the molecular mechanisms through which TSPAN7 influences the adipose tissue biology. To assess the systemic impact of TSPAN7 on whole-body metabolism and adipose tissue distribution, we analyzed the phenotypes of adipose tissue-specific *Tspan7*-knockout and -overexpressing mouse models. Furthermore, to evaluate the translational relevance of our findings, we examined the phenotypes of human adipose-derived stem cells (hADSCs) in an *in vitro* model. An understanding of the role of TSPAN7 in fat distribution could provide crucial insights into obesity pathogenesis and metabolic health, potentially leading to novel therapeutic strategies for obesity and related metabolic diseases.

## 2 Materials and methods

### 2.1 Animal care and experimental design

All experimental procedures were approved and performed in accordance with the Institutional Animal Care and Use Committee of the RIKEN Yokohama Campus and in compliance with the ARRIVE guidelines. Mice were housed in separate cages with a maximum of 5 mice/cage and maintained in an alternating 12 h light/dark cycle at 23 °C with *ad libitum* access to food and water. Mice were fed a standard chow diet (CLEA Rodent Diet CE-2: 12% calories from fat, 59.1% calories from carbohydrates, and 28.8% calories from protein; CLEA Japan Inc, Tokyo, Japan), referred to as the normal diet (ND), or a high-fat diet (HF; CLEA High Fat Diet 32 HFD32: 56.7% calories from fat, 23.1% calories from carbohydrates, and 20.0% calories from protein; CLEA Japan Inc.). The timeline of experiments is presented in Supplementary Fig. S1. Mice were weaned at four weeks of age and fed an ND until they were allocated to either the ND or HF group. The HF group was maintained on HF diet for short (2 weeks), middle (14–18 weeks), and long (26–27 weeks) periods, and mice were euthanized under isoflurane anesthesia. ND-fed mice were sacrificed at different ages: young age (6–10 weeks), early-middle age (16–24 weeks), late middle age (31 weeks), and old age (45–48 weeks). Blood samples were collected, and organs, including inguinal white adipose tissue (iWAT), epididymal white adipose tissue (eWAT), gonadal white adipose tissue (gWAT), brown adipose tissue (BAT), soleus skeletal muscle (muscle), liver, kidney, pancreas, spleen, heart, and lung, were excised, weighed, and submerged in RNAlater solution (Thermo Fisher Scientific, Waltham, MA, USA) at 4 °C for 20 h and stored at −20 °C for a maximum of 6 months.

### 2.2 Mouse models

Conditional knockout mice with a loxP-flanked *Tspan7* allele (*Tspan7*^flox/flox^ mice, targeting exon 5) and conditional overexpression mice with a CAG promoter-loxP-STOP-loxP-*Tspan7* construct at the ROSA26 locus (*Tspan7*^flox/flox^ mice) were crossed with transgenic mice carrying adiponectin (*Adipoq*) promoter-driven Cre recombinase (B6; FVB-Tg(Adipoq-cre)1Evdr/J, Stock#010803, The Jackson Laboratories, Bar Harbor, ME, USA) to produce adipose-specific *Tspan7*-knockout and -overexpressing mice; *Tspan7*^flox/flox^ littermates were used as controls [16]. ES cells for conditional knockout mice with a loxP-flanked *Tspan7* allele (*Tspan7*^flox/flox^ mice, targeting exon 2, Tspan7^tm1a(EUCOMM)Wtsi^) were obtained from European Mouse Mutant Cell Repository (EuMMCR, Neuherberg, Germany). The experimental conditions for comparing C57BL/6J and C57BL/6NCrl mice [29] and those for comparing germ-free mice and fecal microbiota-colonized mice [30] have been described previously.

### 2.3 Cultured cells

hADSCs were purchased from Lonza (Basel, Switzerland) and cultured according to the manufacturer’s instructions until they were approximately 70% confluent. Adipogenic differentiation was induced by treating the cells with an adipogenic induction medium (Lonza), and the medium was refreshed every 2 days. To generate *TSPAN7*-knockdown cells, lentiviral transduction particles were produced from MISSION shRNA plasmid DNA TRC11807 (targeting sequence: GCTTGTTACATACCTGGGTAT) and TRC11809 (targeting sequence: GCATGAACGAAACTGATTGTA) from Sigma–Aldrich (St. Louis, MO, USA). As a control, lentiviral particles were produced using the MISSION pLKO.1-puro Control Vector (SHC001). For *TSPAN7*-overexpressing cells, lentiviral particles were produced using pLenti-C-Myc-DDK-P2A-Puro-TSPAN7 (ORIGENE, Rockville, MD, USA) and pEZ-Lv181-Flag-TSPAN7 (GeneCopoeia, Rockville, MD, USA). The control groups for overexpression experiments used lentivirus particles generated from plasmids containing only a multicloning site without an insert. Lentiviral particles were generated following the manufacturer’s protocol, and hADSCs were transduced with viral particles at a multiplicity of infection optimized for effective transduction. Following transduction, cells were selected and maintained under appropriate antibiotic conditions to ensure stable gene expression. For proteomics experiments, 10 days after adipogenic differentiation, cells were lysed in RIPA buffer and processed for subsequent proteomic analysis. To investigate TSPAN7 localization, hADSCs were transduced with a GFP-tagged TSPAN7 lentivirus generated using the pLV-TSPAN7-GFPSpark plasmid (Sino Biological Inc., Beijing, China). Three days post-transduction, cells were stained with antibodies against LAMP1 (cat#67300-1-IG, 1:50), RAB5B (cat#27403-1-AP, 1:50), RPS3 (cat#66046-1-IG, 1:50), ATP5A (cat#66037-1-IG, 1:150), TGN46 (cat#66477-1-IG, 1:50), PDI (cat#66422-1-IG, 1:50), and CAT (cat#66765-1-IG, 1:50) from Proteintech (Rosemont, IL, USA), ITGB1 (cat#MAB2253, 1:100, EMD Millipore Co., Temecula, CA, USA), and PLIN1 (cat#9349, 1:100, Cell Signaling Technology, Danvers, MA, USA). Secondary labeling was performed using Alexa Fluor 594-conjugaed antibodies (Jackson ImmunoResearch Laboratories, Inc., West Grove, PA, USA). To stain lipid droplets and nucleus, BODIPY (cat#D3922, Thermo Fisher Scientific), LipidTOX (cat#H34476, Thermo Fisher Scientific), and Hoechst 33342 (cat#H1399, Thermo Fisher Scientific) were used according to the manufacture’s recommendations.

Stromal vascular fraction (SVF) from iWAT of *Tspan7*-knockout mice and control littermates was isolated. Minced iWAT was digested with collagenase type I (Sigma–Aldrich) in Dulbecco’s modified Eagle medium (DMEM) at 37 °C for 60 min with gentle shaking. After digestion, the cell suspension was filtered through a 70 μm strainer, and the SVF was collected by centrifugation at 300×*g* for 5 min. The pelleted cells were washed twice with PBS (−) and then resuspended in DMEM containing 10% fetal bovine serum and penicillin/streptomycin for further analysis. SVFs were grown to approximately 80% confluency in a 12-well plate, with and without differentiation. The culture medium was then removed and replaced with serum-free DMEM to which isoproterenol (10 μM) was added. After 2 h of incubation, the cells were washed with cold PBS (−) and lysed using RIPA buffer for western blotting.

### 2.4 Proteomics analysis

Samples were treated with 10 mM dithiothreitol at 50 °C for 30 min and then subjected to alkylation with 30 mM iodoacetamide in the dark at 20–25 °C. The mixture was diluted 4-fold with 50 mM ammonium bicarbonate and digested with 800 ng Lys-C and 400 ng trypsin overnight at 37 °C. An equal volume of ethyl acetate was added to the digested samples, and the mixture was acidified with 0.5% trifluoroacetic acid (TFA) according to the PTS protocols [31,32]. The mixture was shaken for 5 min and centrifuged at 15,000×*g* for 5 min for phase separation; thereafter, the aqueous phase was retrieved. The volume of the digested sample recovered was reduced to half or less of the original volume using a centrifugal evaporator for complete removal of ethyl acetate, and then the mixture was desalted using C18-Stage Tips [33]. The peptides trapped in the C18-Stage Tips were eluted with 40 μL of 50% acetonitrile (ACN) and 0.1% TFA, followed by drying using a centrifugal evaporator. The dried peptides were redissolved in 20 μL of 3% ACN and 0.1% formic acid; 2 μL of the redissolved sample was analyzed via LC-MS/MS.

A preliminary data dependent acquisition (DDA) set was performed for SWATH protein quantification as described below [34]. Peptides (approximately 100 ng) were directly injected onto a 100 μm × 15 cm PicoFrit emitter (New Objective) packed in-house with 120 A porous C18 particles (ReproSil-Pur C18-AQ 1.9 μm; Dr. Maish GmbH) and then separated using 240 min ACN gradient (3–40%, flow rate 300 nL/min) using an Eksigent ekspert nanoLC 400 HPLC system (Sciex). Peptides eluted from the column were analyzed on a TripleTOF 5600+ mass spectrometer. MS1 spectra were collected in the *m/z* range of 400–1200 for 250 ms. The top 25 precursor ions with charge states of 2+ to 5+ that exceeded 150 counts/s were selected for fragmentation with rolling collision energy, and MS2 spectra were collected for 100 ms. A spray voltage of 2100 V was applied. All MS/MS files were searched against the UniProtKB/Swiss-Prot mouse database (Proteome ID: UP000000589, downloaded October 30, 2019; 17069 protein entries), combined with the standard MaxQuant contaminants database (http://www.coxdocs.org/doku.php?id=maxquant:start_downloads.htm), using the ProteinPilot software v. 4.5 with the Paragon algorithm for protein identification [34]. The search parameters were as follows: cysteine alkylation of iodoacetamide, trypsin digestion, and TripleTOF 5600. For the protein confidence threshold, we used the ProteinPilot unused score of 1.3 with at least one peptide with 95% confidence. Proteins and peptides identified as contaminating proteins were excluded. Global false discovery rate for both peptides and proteins was less than 1%. SWATH data independent acquisitions (DIA) were performed employing the same gradient profile used for the DDA experiments as described above. Precursor ion selection was done in the *m*/*z* range of 400–1200, with a variable window width strategy (7–75 Da). Collision energy for each individual SWATH experiment was set at 45 eV, and 80 consecutive SWATH experiments (100–1800 *m*/*z*) were performed, each lasting 36 ms. DIA raw data were analyzed using the SWATH processing embedded in the PeakView software (SCIEX). Peptides from PIG Trypsin and Protease I precursor Lysyl endopeptidase were used for retention time recalibration between runs. The following criteria were used for DIA quantification: peptide confidence threshold 99%, 30 ppm maximum mass tolerance, and 6 min maximum retention time tolerance. Multivariate data analysis was performed using the Markerview software (SCIEX).

### 2.5 Immunoblotting

Cells and tissues were lysed in RIPA buffer and subjected to SDS-PAGE using Mini-Protean TGX precast gels (Bio-Rad Laboratories, Hercules, CA, USA). Proteins were then transferred onto a polyvinylidene fluoride membrane using the Transblot Turbo transfer system (Bio-Rad). The membranes were blocked with 5% skim milk (Bio-Rad) and incubated overnight at 4 °C with primary antibodies. After washing the membranes three times with Tris-buffered saline with Tween 20 (TBS-T), they were incubated with a secondary antibody for 1 h at room temperature. Following four washes with TBS-T, immunoreactive proteins were detected using Chemi-Lumi One (Nacalai Tesque, Kyoto, Japan). The following antibodies were used: Perilipin-1 (PLIN1, cat#9349, 1:1000), hormone-sensitive lipase (HSL, cat#4107, 1:1000), phospo-HSL (Ser 660, cat#4126, 1:1000), and β-Actin (cat#58169, 1:1000) from Cell Signaling Technology, GAPDH (cat#014–25524, 1:1000) from FUJIFILM Wako Pure Chemical Co. (Osaka, Japan), and TSPAN7 (cat# 18695-1-AP, 1:500) from Proteintech.

### 2.6 Plasma parameters

Plasma insulin (Morinaga Institute of Biological Science, Kanagawa, Japan), leptin (R&D Systems, Minneapolis, MN, USA), and adiponectin (Otsuka Pharmaceutical Co., Ltd., Tokyo, Japan) levels were measured using enzyme-linked immunosorbent assay kits. Plasma glucose, triglyceride (TG), total cholesterol (TCHO), high-density lipoprotein cholesterol (HDL), FFAs, alanine aminotransferase (ALT), aspartate transaminase (AST), and lactate dehydrogenase (LDH) levels were measured using reagents from FUJIFILM Wako Pure Chemical Co. Branched-chain amino acids (BCAA) were measured using a BCAA Assay Kit (Cell Biolabs, Inc., San Diego, CA, USA). All assays were performed according to the manufacturer’s instructions. Blood free glycerol (FG) levels (FUJIFILM Wako Pure Chemical Co.) were quantified after isoproterenol (10 mg/kg body weight) was administered to 15-week-old male *Tspan7*-knockout mice and their littermate control mice via intraperitoneal injection.

### 2.7 Glucose tolerance test (GTT) and insulin tolerance test (ITT)

GTT was performed on ND-fed male and female mice at 8 weeks of age and 10-day HF-fed male and female mice at 10 weeks of age after an overnight fasting (13 h). Mice were administered d-glucose (3 g/kg body weight) dissolved in sterile normal saline and administered via oral gavage using a sterile gavage needle. Blood samples were collected at 0 (baseline), 15, 30, 60, 90, 120, and 240 min post-administration. ITT was conducted on ND-fed male and female mice at 9 weeks of age and 10-day HF-fed male and female mice at 11 weeks of age after a 5 h fast. Mice were injected intraperitoneally injected with human insulin (HumulinR 0.75 U/kg body weight, Eli Lilly, Indianapolis, USA). Blood samples were collected at 0 (baseline), 20, 40, 60, 90, and 120 min post-injection. Plasma glucose, insulin, FFAs, and FG levels were measured as described in section 2.6.

### 2.8 Histological analysis

Tissues samples were fixed in 10% formalin (FUJIFILM Wako Pure Chemical Co.) for 20 h, dehydrated in a graded series of sucrose-solutions (10% and 30%), and embedded in Neg-50 medium (Epredia, Breda, Netherlands). Sections (20 μm-thick) were cut using a cryostat microtome and subsequently subjected to hematoxylin and eosin (HE) staining (Meyer’s hematoxylin solution and 1% eosin, Muto Pure Chemicals Co., Ltd., Tokyo, Japan). Images of differentiated hADSCs, following *TSPAN7* knockdown or control treatment, were captured directly in culture. All images were captured using a BZ-X710 microscope (Keyence, Osaka, Japan), and analyzed using BZ-X Viewer version 1.3.1.1 (Keyence) and BZ-X Analyzer version 1.3.1.1 (Keyence).

### 2.9 Tissue and cultured cell preparation and RNA isolation

Minced tissues were homogenized in Sepasol RNAI solution (Nacalai Tesque, Kyoto, Japan) using a TissueLyser LT instrument (Qiagen, Hilden, Germany) set at 50 strokes/s for 5 min. The adipose tissue homogenate was centrifuged at 3,000 *× g* for 10 min to separate fat layer, and the bottom layer was transferred to a new tube. Chloroform was added and the vortexed sample was centrifuged at 14,000 *× g* for 10 min. The RNA phase was transferred to a fresh tube for total RNA purification. For cultured cells, ADSCs were harvested after 20 days of culture and lysed in RLT buffer (Qiagen). The lysate was further disrupted using QIAshredder spin column (Qiagen) before total RNA purification. Total RNA was purified using QIAcube and the RNeasy kit (Qiagen). RNA quality was assessed using a TapeStation system (Agilent Technologies, Santa Clara, CA, USA) with RNA ScreenTape (Agilent).

### 2.10 Library construction and sequencing

Libraries were constructed using the NEBNext Ultra II RNA Library Prep Kit for Illumina (New England Biolabs, Ipswich, MA, USA). The mRNA was enriched from the total RNA (250 ng) using magnetic poly-T beads. First- and second-strand cDNAs were synthesized using random hexamer primers (included in the kit), M-MuLV reverse transcriptase, DNA polymerase I, and RNase H, followed by the conversion of overhangs to blunt ends. DNA fragments were ligated with NEBNext adaptors and size-fractionated using the AMPure XP system (Beckman Coulter, Inc., Brea, CA, USA) before treatment with USER enzyme (New England Biolabs). PCR amplification was performed with universal and index primers using Phusion high-fidelity DNA polymerase. The PCR products were purified with the AMPure XP system, and the quality of the library was assessed using the TapeStation system (Agilent). Pooled libraries were sequenced on Illumina NextSeq 2000 to obtain 50 bp single-end reads.

### 2.11 Read mapping and differentially expressed gene (DEG) analysis

Reads were aligned and mapped to genes in the reference mouse genome (UCSC mm10) and the human genome (GRCh37/hg19), and assembled into transcripts using StrandNGS (v.4.0, Strand Life Sciences, Bangalore, India). Normalized gene expression values in transcripts per kilobase million were used to compare sample group pairs that included at least three biological replicates per group. The significance of the differences in gene expression levels between the groups was analyzed using the unpaired Mann–Whitney U-test (45,796 genes in total, absolute fold change >1.0, and adjusted *p*-value <0.05).

### 2.12 cDNA synthesis and qPCR analysis

First strand cDNA was synthesized using a ReverTra Ace qPCR RT Kit (Toyobo, Shiga, Japan) following the manufacturer’s instructions. q-PCR was performed using a LightCycler96 (Roche, Basel, Switzerland) in a reaction containing THUNDERBIRD Next SYBR qPCR Mix (Toyobo) with the following primers. *Tspan7*, (forward) 5′-TTGGATGCTTTGCTACATGC-3′ and (reverse) 5′-AATCCAGAAATGCCAGCAAC-3′; *Actb*, (forward) 5′-TTCTTTGCAGCTCCTTCGTT-3′ and (reverse) 5′-ATGGAGGGGAATACAGCCC-3′; *Bckdhb*, (forward) 5′-CTTCCGATGCACTGTTGGTTT-3′ and (reverse) 5′-GATTTCCGCAATAGCTGTAGCA-3′; *Hprt*, (forward) 5′-CACAGGACTAGAACACCTGC-3′ and (reverse) 5′-GCTGGTGAAAAGGACCTCT-3′.

### 2.13 Statistical analysis

Statistical analyses were performed using GraphPad Prism 8 (GraphPad Software, San Diego, CA, USA) and SPSS 29.0 (SPSS Inc., Armonk, NY, USA). The normality and homogeneity of variance of the data were verified using the Shapiro–Wilk test and Levene’s test, respectively. If a normal distribution could not be assumed, the nonparametric Mann–Whitney *U*-test was performed. In cases of unequal variance, we applied Welch’s correction test. To assess differences between samples, we employed a one-way analysis of variance (ANOVA) followed by Dunn’s multiple comparison test. To determine the statistical difference in tissue weight gain between the two groups, we performed a simple linear regression analysis with age as a covariate. Significance levels between groups were represented as ∗*p* < 0.05, ∗∗*p* < 0.01, ∗∗∗*p* < 0.001, ∗∗∗∗*p* < 0.0001, ^†^, *p* < 0.05; ^††^, *p* < 0.01; ^†††^, *p* < 0.001; ^††††^, *p* < 0.0001; for 0.05 < *p* < 0.1, the absolute *p*-value was noted. To evaluate the correlation between eWAT/iWAT and glucose/insulin or BCAA concentration, Pearson’s correlation coefficients were calculated. GTT and ITT data were analyzed using repeated measures ANOVA based on a general linear model with the Geisser-Greenhouse correction. Multiple comparisons between group means were conducted using Bonferroni method [35].

## 3 Results

### 3.1 *Tspan7* expression in adipose tissue is associated with metabolic changes

We first examined the effect of diet-induced weight gain over time on *Tspan7* expression in various tissues using female mice fed either an ND or HF for 20 weeks, starting at 7 weeks of age (Figure 1A). *Tspan7* expression in iWAT, gWAT, and the heart was significantly decreased (*p* < 0.01) after 4 weeks of HF consumption compared with that in age-matched ND-fed mice, and the reduced expression levels were maintained throughout the examined period. Additionally, a tendency for decreased *Tspan7* expression with HF intake was observed in BAT, muscle, and pancreas, although the changes were less pronounced than those in white adipose tissue (WAT). Moreover, *Tspan7* expression was notably high in mitochondria-rich tissues, such as the heart, muscle, and kidneys, and immunofluorescence analysis revealed its localization to mitochondria in hADSCs (Supplementary Fig. S2). TSPAN7 was also found to localize around lipid droplets in differentiated hADSCs, as demonstrated by staining with LipidTOX (Supplementary Fig. S3). No notable changes in *Tspan7* expression were observed in the brain, liver, lung, small intestine, kidney, spleen, and stomach upon HF intake. No statistically significant age-dependent changes were observed in any tissues of ND-fed mice examined up to 27 weeks of age. These findings suggest that *Tspan7* expression in WAT is particularly sensitive to diet-induced obesity. To further investigate the potential involvement of *Tspan7* in WAT and its association with body weight regulation, we analyzed *Tspan7* expression in different mouse models with distinct body weight regulation patterns.

**Figure 1:**
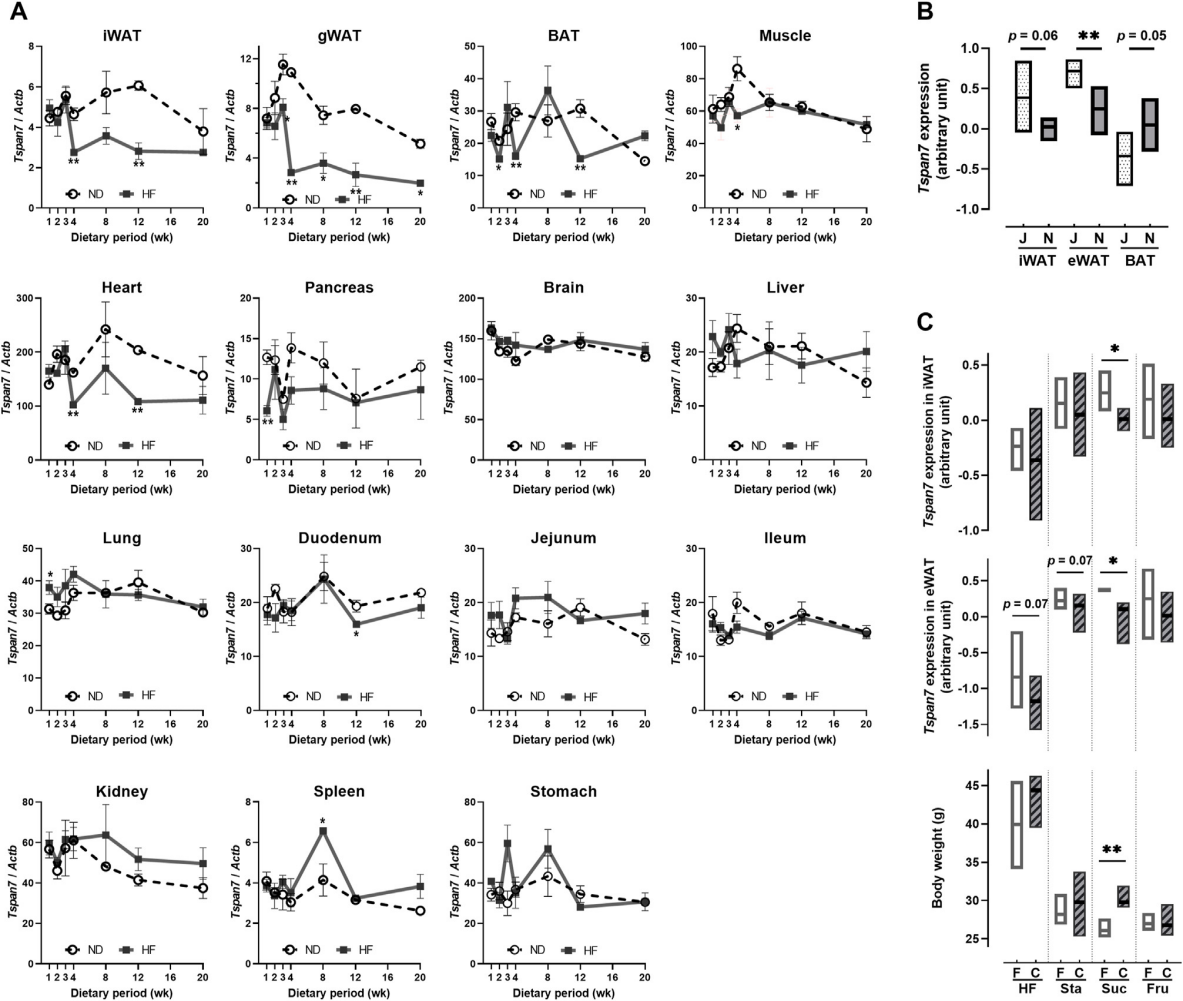
***Tspan7* expression in adipose tissue is associated with metabolic changes.** (A) *Tspan7* expression in iWAT, gWAT, BAT, muscle, heart, pancreas, brain, liver, lung, duodenum, jejunum, ileum, kidney, spleen, and stomach, of female C57BL/6J mice fed a normal diet (ND; open circles) or high-fat diet (HF; closed circles) diet for 20 weeks, starting at 7 weeks of age. Gene expression was measured using quantitative PCR and normalized against β-actin (*Actb*) expression. Bars represent mean ± SEM (*n* = 3–7 per group). Statistical significance was determined using a two-tailed unpaired *t*-test. ∗, *p* < 0.05 between ND- and HF-fed groups within the same age groups. (B) *Tspan7* expression in iWAT, eWAT, and BAT of ND-fed male C57BL/6J (J; dotted box) and C57BL/6N (N; filled box) mice. Data are shown as whisker plots, from minimum to maximum, with mean bars (*n* = 5 per group). ∗∗, *p* < 0.01 between J and N groups within the same tissue groups. (C) *Tspan7* expression in iWAT and eWAT of male germ-free (F; open box) and germ-colonized (C; slashed box) mice fed HF, high-starch (Sta), high-sucrose (Suc), and high-fructose (Fru) diets for 8 weeks, starting at 7 weeks of age. Data are shown as whisker plots, from minimum to maximum, with mean bars (*n* = 4–7 per group). ∗, *p* < 0.05; ∗∗, *p* < 0.01 between F and C groups under the same dietary conditions. iWAT, inguinal white adipose tissue; gWAT, gonadal white adipose tissue; eWAT, epididymal white adipose tissue; BAT, brown adipose tissue; muscle, skeletal soleus muscle; SEM, standard error of the mean.

Figure 1B presents the results of a comparison of *Tspan7* expression in iWAT, eWAT, and BAT between two mouse strains, C57BL/6J (6J) and C57BL/6N (6N), which differ in their susceptibilities to weight gain [29]. *Tspan7* expression in the iWAT and eWAT of 38-week-old ND-fed obesity-prone male 6N mice was significantly lower (*p* = 0.06 and *p* < 0.01, respectively) than that in the obesity-resistant male 6J mice of the same age. Furthermore, Figure 1C presents a comparative illustration of *Tspan7* expression in iWAT and eWAT between germ-free mice, known to exhibit suppressed weight gain, and germ-colonized mice. Eight-week-old male mice were fed high-fat, high-starch, high-sucrose, and high-fructose diets for 8 weeks. Regardless of the diet, *Tspan7* expression in iWAT and eWAT was higher in germ-free mice. Among all diet conditions, the sucrose diet group showed the most pronounced difference in *Tspan7* expression (*p* < 0.05) between germ-free and germ-colonized mice, which was reflected in body weight differences (*p* < 0.01). These results suggest that TSPAN7 may play a role in adipose tissue function, which in turn influences body weight regulation.

### 3.2 *Tspan7* may play a role in lipid droplet dynamics

To further investigate the role of TSPAN7 in adipose tissue, we examined the morphology of adipocytes in *Tspan7*-knockout and *Tspan7*-overexpressing mice, where *Tspan7* expression was specifically manipulated in adipose tissue via Adipoq-Cre. HE-stained images of iWAT from 20-week-old male *Tspan7*-knockout mice fed HF for 15 weeks showed smaller adipocyte sizes than those in the littermate controls (Figure 2A). Figure 2B shows the quantification of these differences, including the absolute weights of iWAT and e(g)WAT and their percentage relative to body weight, adipocyte area, and frequency of adipocytes per area in male and female *Tspan7*-knockout mice. No significant differences in iWAT and e(g)WAT weights were noted between the two groups. However, the adipocyte area was significantly smaller (*p* < 0.001), and the frequency of smaller adipocytes was higher in male and female knockout mice than that in littermate controls. Similarly, in *Tspan7*-overexpressing males and females (Supplementary Fig. S4), no significant differences in iWAT and e(g)WAT weights were noted between the two groups. However, the adipocyte area was significantly smaller (*p* < 0.001), and the frequency of smaller adipocytes was higher in male iWAT and female iWAT and gWAT than that in littermate controls (Supplementary Fig. S4). Furthermore, when considering the effects of ND, 20-week-old ND-fed knockout and overexpressing mice also exhibited smaller adipocyte sizes in iWAT than that in the respective littermate controls (Supplementary Fig. S5). In contrast, no significant difference in adipocyte sizes in BAT was observed between HF-fed knockout or overexpressing mice and their respective controls (Supplementary Fig. S6).

**Figure 2:**
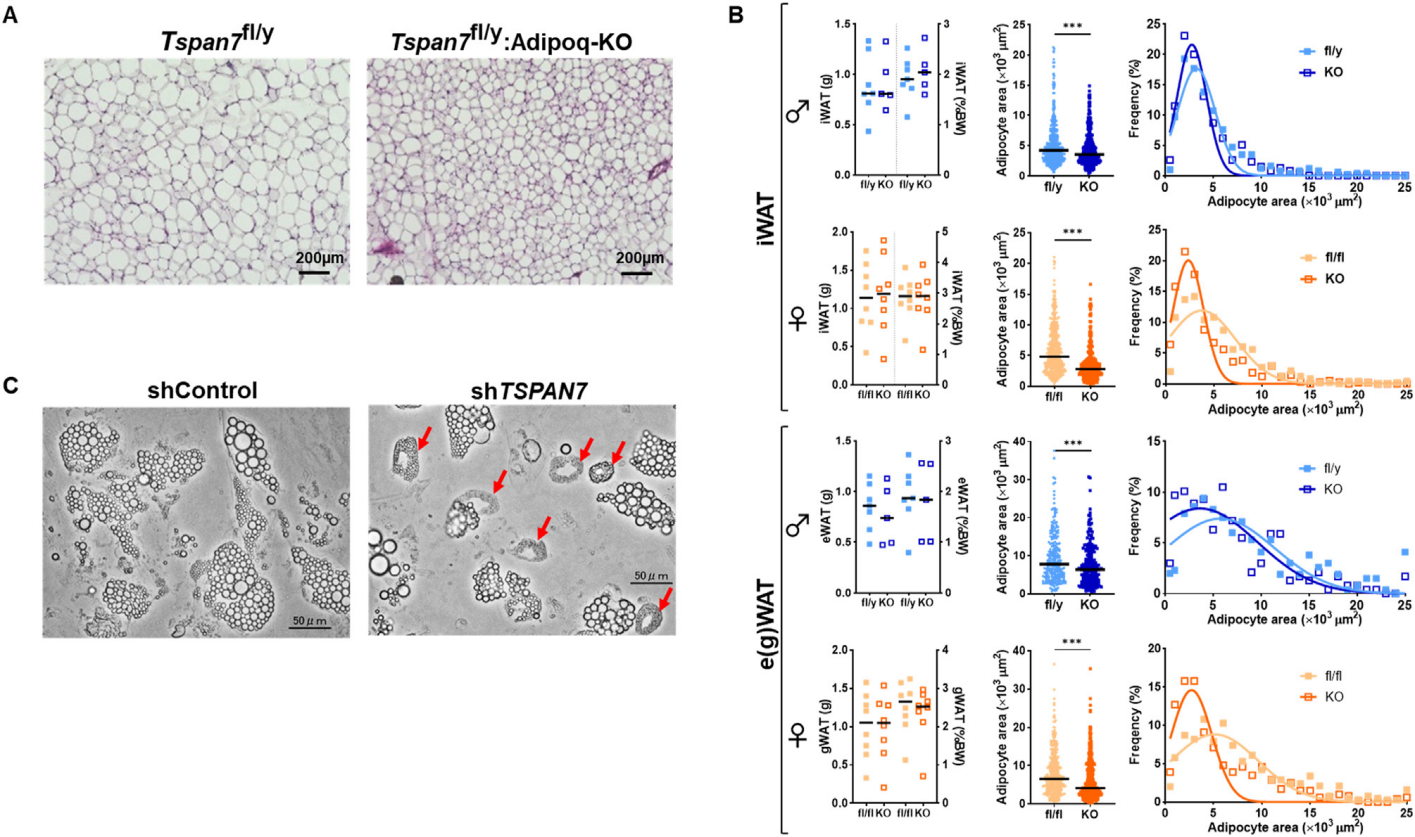
***Tspan7* may play a role in lipid droplet dynamics.** (A) Representative HE-stained images of iWAT from 20-week-old male *Tspan7*-knockout (*Tspan7*^fl/y^; Adipoq-KO) and littermate (*Tspan7*^fl/y^) mice fed HF for 15 weeks. Scale bar: 200 μm. (B) Absolute weight and body-weight percentage (left panels, squares indicate individual data points), adipocyte area (middle panels, 450–970 cells counted per group), and frequency distribution of adipocyte area (right panel, Gaussian least-squares fit) in iWAT and e(g)WAT of 20-week-old male (blue) and 21-week-old female (orange) *Tspan7*-knockout (KO, open squares) and littermate (fl/y for males, fl/fl for females, filled squares) mice. Bars represent the median (*n* = 10–14 per group). Statistical significance was assessed using the Mann–Whitney *U*-test. ∗∗∗, *p* < 0.001 between fl/y(fl) and KO. (C) Representative images of adipocytes differentiated from hADSCs with *TSPAN7* knockdown (shTSPAN7, right panel) and control (shControl, left panel). Donut-shaped adipocytes, with clusters of small lipid droplets, are indicated by red arrows. Scale bar: 50 μm. HE, hematoxylin and eosin; HF, high-fat diet; iWAT, inguinal white adipose tissue; eWAT, epididymal white adipose tissue; gWAT, gonadal white adipose tissue; hADSCs, human adipose tissue-derived stem cells. (For interpretation of the references to colour in this figure legend, the reader is referred to the Web version of this article.)

To further support the relevance of these findings in human cells, we examined the effects of TSPAN7 on adipocyte morphology in hADSCs. Figure 2C shows images of adipocytes differentiated from hADSCs. Donut-shaped adipocytes, with clusters of very small lipid droplets, were observed upon *TSPAN7* knockdown using short hairpin RNA (shRNA), distinct from the larger lipid droplets seen in adipocytes transduced with a control shRNA. These adipocytes were stained with BODIPY to confirm the presence of lipid droplets (Supplementary Fig. S7). This type of adipocyte accounted for approximately 40% of the total adipocytes differentiated from *TSPAN7*-knockdown hADSCs. These results indicate that TSPAN7 may be involved in the regulation of lipid droplet formation in human cells.

### 3.3 TSPAN7 may modulate molecular pathways involved in lipid droplet formation and metabolic processes

To explore the molecular mechanisms underlying these findings, we performed a proteomic analysis to identify the molecules affected by altered TSPAN7 levels (Figure 3A–C). The proteomic profiles of two clones each of hADSCs with *TSPAN7* knockdown or overexpression and their respective controls were compared. Of the 1,979 proteins detected, 732 exhibited higher expression levels and 674 exhibited lower expression levels in two *TSPAN7*-knockdown cell clones compared with the control cells. Similarly, 730 exhibited higher expression levels and 609 exhibited lower expression levels in two *TSPAN7*-overexpressing cell clones compared with those in the control cells (Figure 3A).

**Figure 3:**
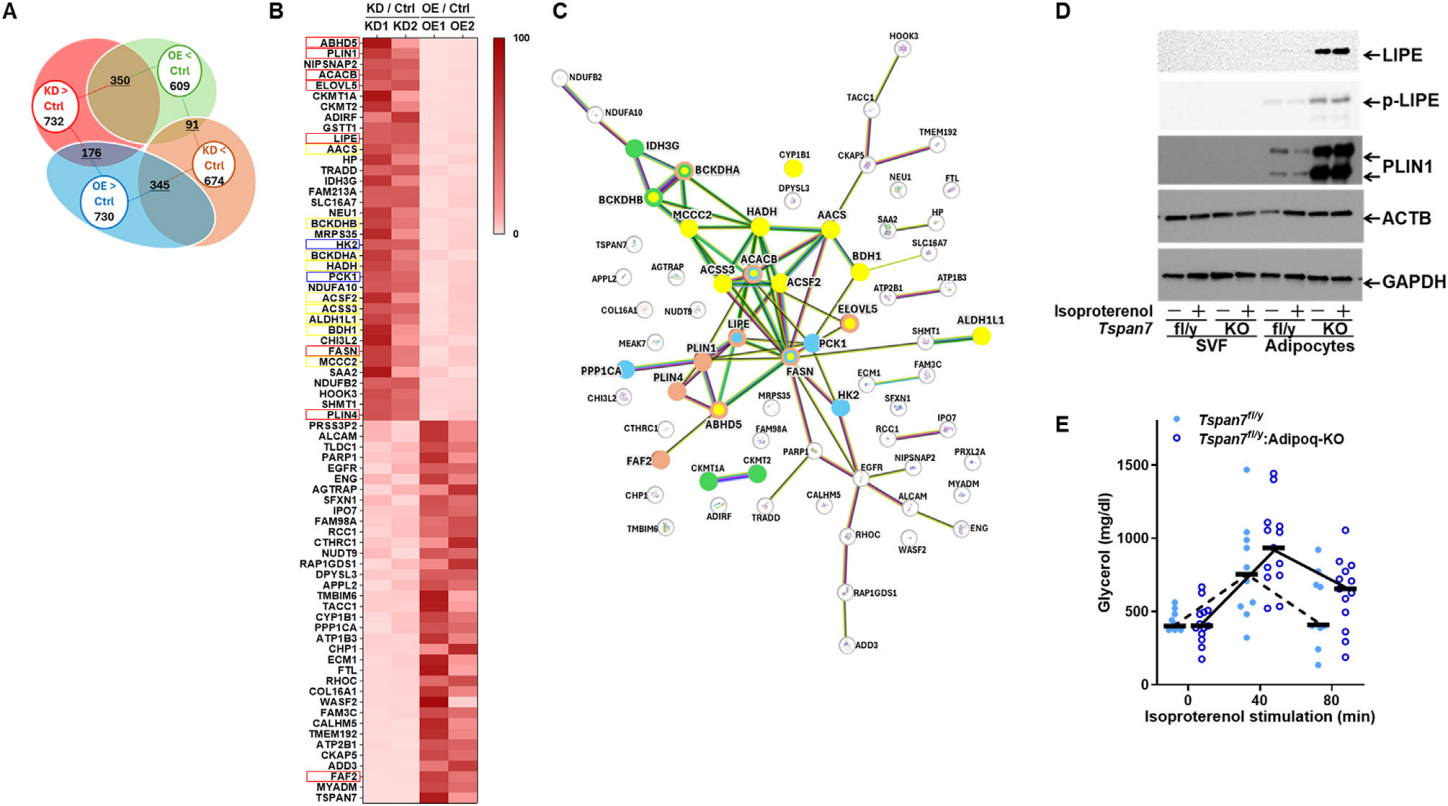
***Tspan7* may modulate molecular pathways involved in lipid droplet formation and metabolic processes.** (A) Venn diagram showing the number of differentially expressed proteins in hADSCs upon *TSPAN7* knockdown (KD) or overexpression (OE) compared to their respective controls (Ctrl). Proteins are categorized into four groups: KD > Ctrl, OE > Ctrl, KD < Ctrl, and OE < Ctrl. Numbers within circles indicate the count of proteins in each category. (B) Heatmap of selected differentially expressed proteins. Thirty proteins meeting the KD > Ctrl and OE < Ctrl criteria, and thirty proteins meeting the KD < Ctrl and OE > Ctrl criteria, were visualized. Data reflect consistency for two independent KD and OE clones. (C) Protein–protein interaction networks generated using STRING database analysis of the 60 proteins shown in (B). Based on term descriptions: proteins associated with “lipid droplet” are shown in pink, “fatty acid” in yellow, “mitochondrion” in green, and “insulin signaling” in blue. (D) Western blot analysis of PLIN1, LIPE, p-LIPE, ACTB, and GAPDH expression in SVF-derived adipocytes. SVFs were isolated from iWAT of *Tspan7*-knockout mice, differentiated into adipocytes, and treated with isoproterenol (1 μM) for 20 min. (E) Plasma free glycerol levels in male *Tspan7*-knockout mice (*Tspan7*^fl/y^:Adipoq-KO, open circles) and their littermate controls (Tspan7^fl/y^, filled circles) at 40 and 80 min after i.p. isoproterenol injection (1 mg/kg body weight). Circles indicate individual data points (*n* = 10–13 per group). hADSCs, human adipose-derived stem cells; SVF, stromal vascular fraction; iWAT, inguinal white adipose tissue; PLIN1, perilipin 1; LIPE, hormone-sensitive lipase; p-LIPE, phosphorylated LIPE; ACTB, beta-actin; GAPDH, glyceraldehyde-3-phosphate dehydrogenase; i.p., intraperitoneal. (For interpretation of the references to colour in this figure legend, the reader is referred to the Web version of this article.)

A total of 350 proteins showed increased expression with *TSPAN7* knockdown and decreased expression with *TSPAN7* overexpression, whereas 345 proteins exhibited the opposite pattern, increasing with *TSPAN7* overexpression and decreasing with *TSPAN7* knockdown (Figure 3A). Of these, the top 30 proteins with the most significant changes in expression (fold change: knockdown/control or overexpression/control) were listed and visualized using a heatmap (Figure 3B). These 60 proteins were then subjected to protein–protein interaction analysis using the STRING database, which revealed a network of proteins associated with lipid metabolism, including lipid droplets, fatty acid metabolism, mitochondrion, and insulin signaling (Figure 3C and Table S1). The protein with the highest node degree was fatty acid synthase (FASN, degree = 15, Supplementary Table S1), which is involved in fatty acid metabolism, lipid droplet formation, and insulin signaling. The next highest was acetyl-CoA carboxylase beta (ACACB, degree = 9, Supplementary Table S1), which is also involved in these three processes. Hydroxyacyl-CoA dehydrogenase (HADH), which also had a high node degree (9, Table S1), is involved in fatty acid metabolism. Lipid droplet-related proteins, such as lipase, hormone sensitive (LIPE) and perilipin 1 (PLIN1), exhibited high node degrees (8 and 7, respectively; Supplementary Table S1) and were incorporated into the network (Figure 3C). In the STRING cluster analysis, FASN, LIPE, PLIN1, perilipin 4 (PLIN4), alpha/beta hydrolase domain-containing 5 (ABHD5), ACACB, and elongation of very long chain fatty acids protein 5 (ELOVL5) showed high strength (1.46) and a low false discovery rate (FDR, 4.2E-05) in the cluster “CL:9985, Fatty Acid Metabolism and Regulation of Lipid Storage,” suggesting the possible involvement of TSPAN7 in lipid metabolism. Additionally, the association with lipid droplet-related pathways was indicated in the UniProt Keyword analysis (KW0551, strength 1.42, FDR 0.0031), COMPARTMENTS analysis (GOCC:0005811, strength 1.27, FDR 0.0019), and gene ontology (GO) Component analysis (GO:0005811, strength 1.15, FDR 0.0059) (Supplementary Table S1). Additionally, the transcriptional levels of these differentially expressed proteins were consistent with these proteomic changes (Supplementary Fig. S8A). This concordance between transcriptomic and proteomic data further supports the notion that TSPAN7 regulates lipid metabolism. Furthermore, we examined the expression levels of genes associated with the GO terms “lipid droplet” (related to lipid droplet dynamics), “fat cell differentiation” (associated with adipogenesis), “lipid catabolic process” (involved in lipolysis), and “mitochondrion” and found that most of these genes also showed noticeable changes upon *TSPAN7* knockdown (Supplementary Fig. S8B).

To investigate the role of TSPAN7 in the lipid droplet synthesis pathway, we measured the levels of PLIN1 and LIPE proteins in SVF cells isolated from the iWAT of *Tspan7*-knockout male mice and their littermate controls. We assessed these proteins in adipocytes differentiated from these SVF cells in culture, with and without isoproterenol treatment (Figure 3D). The expression of PLIN1 and LIPE, key regulators of lipid droplet synthesis, was markedly higher in adipocytes from knockout mice than in those from littermate controls. To evaluate whether these cellular changes translated to systemic effects, we measured blood glycerol levels following isoproterenol administration (Figure 3E). Isoproterenol stimulates protein kinase A via β-adrenergic receptors, leading to the phosphorylation of lipolysis-related proteins, such as PLIN1 and LIPE, which in turn promotes the breakdown of TGs and the release of FFAs and glycerol into the bloodstream. In *Tspan7*-knockout male mice, blood glycerol levels at 40 min post-isoproterenol injection exhibited a greater increase compared to littermate controls, suggesting enhanced lipolysis (Figure 3E). However, despite these systemic effects, no clear differences in PLIN1 and LIPE phosphorylation were observed in differentiated adipocytes, even after isoproterenol treatment (Figure 3D). This discrepancy suggests that while TSPAN7 may influence systemic lipolysis, its role in regulating lipolysis-related protein activation at the cellular level remains unclear.

In line with this discrepancy, gene expression analysis of iWAT from *Tspan7*-knockout and -overexpressing mice did not reveal notable differences in the transcriptional levels of differentially expressed proteins identified in the proteomic analysis (Figure 3C), including lipid droplet-associated genes (Figure 4A) and other differentially expressed proteins (Supplementary Fig. S9). Similarly, genes involved in lipid droplet dynamics, adipogenesis, and lipolysis also showed no apparent changes in gene expression *in vivo* (Supplementary Fig. S10–S12). Thus, despite the clear TSPAN7-dependent changes in the expression of lipid metabolism-related genes observed at the cellular level, these changes were largely absent in adipose tissue. Notably, however, one gene exhibited a consistent expression pattern between hADSCs and adipose tissue—branched-chain keto acid dehydrogenase E1 subunit beta (BCKDHB). In *TSPAN7*-knockdown hADSCs, *BCKDHB* expression was higher than in control cells (Supplementary Fig. S8A). Similarly, in *Tspan7*-modified mice, *Bckdhb* expression was significantly elevated in both *Tspan7*-knockout (*p* < 0.05, both males and females) and -overexpressing (*p* < 0.0001, both males and females) mice under ND conditions (Figure 4B). Importantly, under HF-feeding conditions, *Bckdhb* expression exhibited a *Tspan7* expression-dependent pattern: it decreased in knockout mice but increased in overexpressing mice (Figure 4B). The increase in *Bckdhb* expression in *Tspan7*-modified mice under ND conditions was also observed in eWAT (*p* < 0.05, both males and females, Figure 4C), liver (*p* < 0.05, both males and females, Figure 4D), and muscle (*p* < 0.01, Figure 4E), but not in the pancreas. Because *Tspan7* expression was specifically manipulated in adipose tissue and remained unaltered in liver and muscle, these results suggest that *Tspan7* may modulate *Bckdhb* expression through adipose tissue-derived signals, potentially influencing systemic metabolic processes.

**Figure 4:**
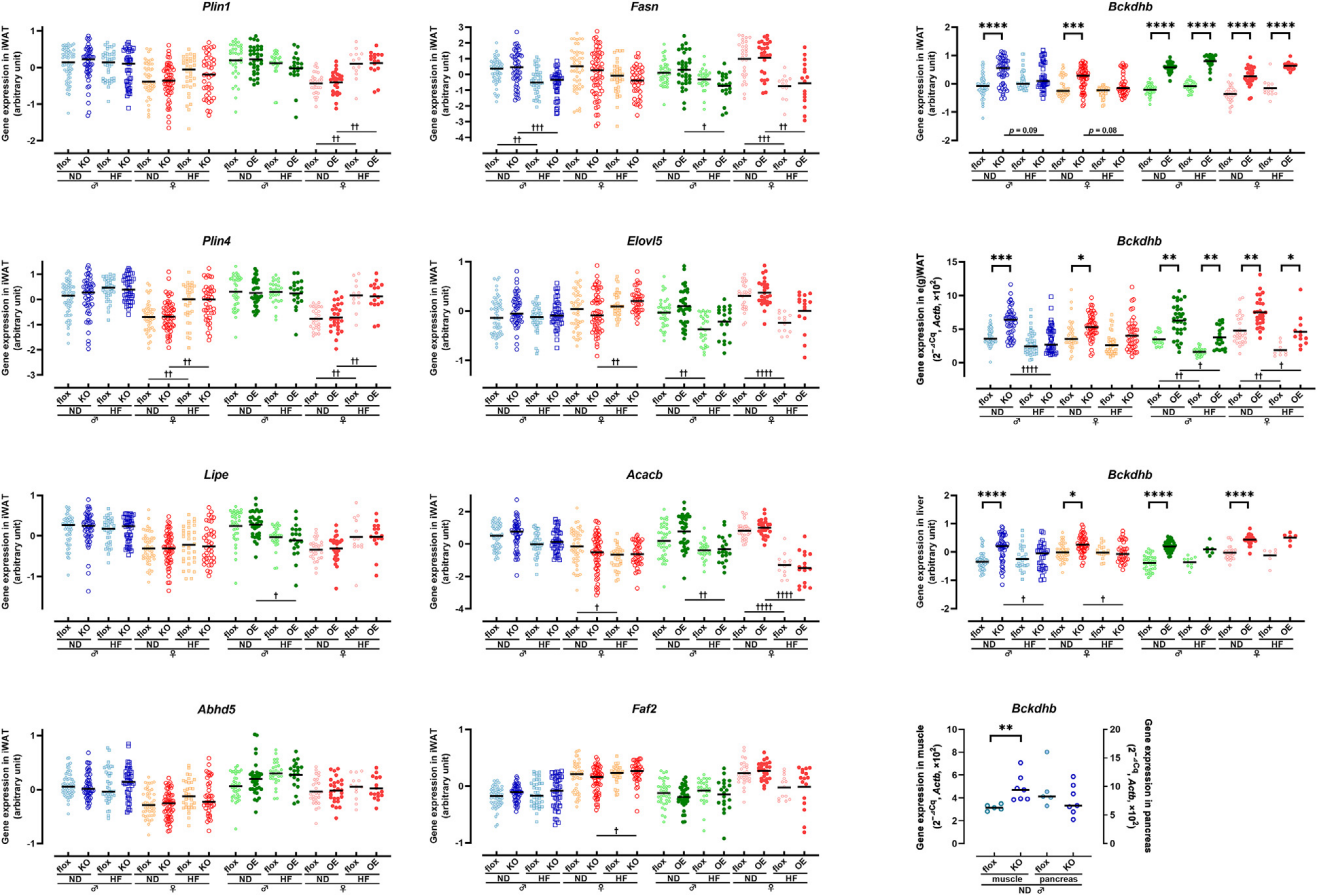
**Gene expression analysis of *Tspan7*-modified mice reveals the metabolic role of TSPAN7.** (A) Expression levels of lipid droplet-associated genes, including *Plin1*, *Plin4*, *Lipe*, *Abhd5*, *Fasn*, *Elovl*5*, Acacb*, and *Faf2* in iWAT. (B–E) Expression levels of *Bckdhb* in (B) iWAT, (C) e(g)WAT, (D) liver, and (E) muscle and pancreas. Data are shown for *Tspan7*-KO male (blue) and female (orange), OE male (green) and female (red), and the respective control mice (light blue, light orange, light green, and pink). Mice were fed a normal diet (ND, circles) or a high-fat diet (HF, squares). Each symbol represents an individual data point, and bars indicate the median (*n* = 5–31 per group). Gene expression levels were measured using RNA sequencing or qPCR. Statistical significance was determined using the Kruskal–Wallis test. Comparisons between KO (or OE) and flox control groups are indicated as ∗, *p* < 0.05; ∗∗, *p* < 0.01. Comparisons between ND and HF groups are indicated as ^†^, *p* < 0.05; ^††^, *p* < 0.01; ^†††^, *p* < 0.001; ^††††^, *p* < 0.0001. iWAT, inguinal white adipose tissue; *Plin1*, perilipin 1; *Plin4*, perilipin 4; *Lipe*, hormone-sensitive lipase; *Abhd5*, alpha/beta hydrolase domain-containing 5; *Fasn*, fatty acid synthase; *Elovl5*, ELOVL fatty acid elongase 5; *Acacb*, acetyl-CoA carboxylase beta; *Faf2*, FAS-associated factor 2; *Bckdhb*, branched-chain α-keto acid dehydrogenase complex subunit B; KO, knockout; OE, overexpressing. (For interpretation of the references to colour in this figure legend, the reader is referred to the Web version of this article.)

### 3.4 TSPAN7 may influence insulin-related metabolic processes

To further investigate how TSPAN7 influences systemic metabolic processes, we analyzed various blood markers associated with lipid regulation, including insulin, leptin, adiponectin, glucose, FFA, TG, TCHO, HDL, AST, ALT, and LDH in *Tspan7*-knockout (Figure 5A–K) and *Tspan7*-overexpressing mice (Supplementary Fig. S13 A–K). We compared baseline values (at a young age, 6–10 weeks), values at an older age (34–38 weeks), and values under short-term (2 weeks) and long-term (22–30 weeks) HF conditions, separately for males and females. At a young age, *Tspan7* modification had minimal effects on these blood markers, with the exception of glucose concentration, which was significantly lower in male *Tspan7*-knockout mice than that in controls (*p* < 0.05, Figure 5A). Following 2 weeks of HF feeding, blood glucose concentrations significantly increased in both control and knockout males (both *p* < 0.001), whereas those in *Tspan7*-knockout mice remained significantly lower than those in control mice (*p* < 0.05). A similar trend was observed in female *Tspan7*-knockout mice, leading to a significant difference (*p* < 0.01) under short-term HF feeding conditions.

**Figure 5:**
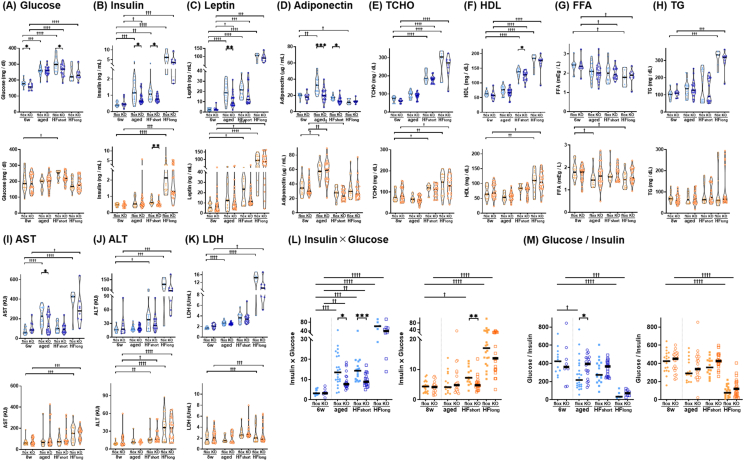
***Tspan7* may influence insulin-related metabolic processes.** Metabolic parameters in the plasma of male (blue) and female (orange) *Tspan7*-KO mice (open circles) and littermate controls (flox, filled circles) fed an ND (baseline, 6–8 weeks; aged, 34–38 weeks) or HF for different periods (short, 2 weeks; long, 22–30 weeks). (A) Glucose, (B) insulin, (C) leptin, (D) adiponectin, (E) TCHO, (F) HDL, (G) FFA, (H) TG, (I) AST, (J) ALT, (K) LDH, (L) insulin-glucose product, and (M) glucose-insulin ratio. For each box plot, the central mark indicates the median, and symbols represent individual data points (*n* = 20–28 per group). Statistical significance was assessed using the Mann–Whitney *U*-test. ∗, *p* < 0.05; ∗∗, *p* < 0.01; ∗∗∗, *p* < 0.001 between flox and KO. ^†^, *p* < 0.05; ^††^, *p* < 0.01; ^†††^, *p* < 0.001; ^††††^, *p* < 0.0001 between the baseline and other time points within the same genotype. KO, knockout; ND, normal diet; HF, high-fat diet; TCHO, total cholesterol; HDL, high-density lipoprotein cholesterol; FFA, free-fatty acids; TG, triglyceride; AST, aspartate aminotransferase; ALT, alanine aminotransferase; LDH, lactate dehydrogenase. (For interpretation of the references to colour in this figure legend, the reader is referred to the Web version of this article.)

In parallel with glucose, insulin levels also responded to HF feeding. Two weeks of HF feeding led to an increase in blood insulin concentrations in both the groups; however, the magnitude of the increase was smaller in *Tspan7*-knockout mice than that in controls. As a result, after short-term HF feeding, blood insulin concentrations were significantly lower in *Tspan7*-knockout mice than those in controls for both males and females (*p* < 0.05 and *p* < 0.01, respectively, Figure 5B). Aging also induced an increase in insulin levels, but again, the extent of this increase was smaller in knockout mice. Consequently, at older ages, blood insulin concentrations were significantly lower in male *Tspan7*-knockout mice than those in controls (*p* < 0.05), whereas no significant difference was observed in females (Figure 5B).

Beyond glucose and insulin, other blood components also exhibited smaller changes in response to aging or HF feeding in *Tspan7*-knockout mice than in controls. In males, these included leptin (aging, *p* < 0.01, Figure 5C), adiponectin (aging, *p* < 0.001; short HF feeding, *p* < 0.05, Figure 5D), HDL (short HF feeding, *p* < 0.05, Figure 5F), and AST (aging, *p* < 0.05, Figure 5I).

In contrast, although *Tspan7*-overexpressing mice exhibited significant differences in adiponectin (short HF feeding in males, *p* < 0.01; aging in females, *p* < 0.05, Supplementary Fig. S13D), TCHO (long HF feeding in males and females, *p* < 0.01 and *p* < 0.05, respectively, Supplementary Fig. S13E), HDL (long HF feeding in males, *p* < 0.05, Supplementary Fig. S13F), and FFA (long HF feeding in males, *p* < 0.05, Supplementary Fig. S13G), unlike in knockout mice, these differences were not consistently associated with aging or HF feeding and could not be systematically explained. The adipose tissue specificity of our genetic modifications was confirmed, with comparable decreases and increases in TSPAN7 protein levels in KO and OE mice, respectively (Supplementary Fig. S14A and B). Notably, while *Tspan7* mRNA expression was significantly altered in both models, the increase in OE mice was more pronounced than the decrease in KO mice (Supplementary Fig. S14C and D). However, the asymmetry in metabolic responses suggests that the differential metabolic outcomes between OE and KO mice are unlikely to be attributed to differences in the magnitude of TSPAN7 expression changes.

Given the reduced insulin elevation observed in *Tspan7*-knockout mice during metabolic fluctuations, we next assessed insulin responsiveness by evaluating insulin resistance and insulin sensitivity using the insulin-glucose product levels and the glucose:insulin ratio. In males, insulin-glucose product levels were significantly lower in knockout mice than those in controls at an older age (*p* < 0.05, Figure 5L) and after short-term HF feeding (*p* < 0.001, Figure 5L). Additionally, the glucose:insulin ratio was significantly higher in knockout mice at an older age than that in controls (*p* < 0.05, Figure 5M). In females, significantly lower insulin-glucose product levels were observed in knockout mice after short-term HF feeding (*p* < 0.01, Figure 5L), with a trend toward higher glucose:insulin ratios in knockout mice at an older age and after HF feeding (Figure 5M).

To further investigate this possibility, we conducted ITT (Fig. S15A) and GTT (Fig. S15B), along with the FFA and free glycerol (FG) analysis. In both tests, knockout mice exhibited an appropriate reduction in blood glucose levels, with no consistent or significant differences compared to controls, leaving the role of TSPAN7 in glucose clearance unclear. However, a difference emerged when assessing lipid metabolism. The reduction in circulating FFA and FG following insulin administration was greater in *Tspan7*-knockout mice than in controls (Fig. S15A). This effect was observed both when insulin was administered exogenously (ITT, Fig. S15A) and when it was endogenously stimulated by glucose administration (GTT, Fig. S15B). The results of the statistical analysis are summarized in Table S2. These findings suggest that TSPAN7 deletion enhances the suppressive effect of insulin on lipolysis and increases insulin sensitivity in adipose tissue, further supporting the role of TSPAN7 in regulating insulin responsiveness, particularly in lipid metabolism.

### 3.5 TSPAN7 may be involved in the regulation of fat-storage sites

Given the close link between insulin action and adipose tissue function, we next investigated whether TSPAN7 influences adipose tissue mass and distribution. The amounts of subcutaneous fat (absolute iWAT: Figure 6A, body-weight percentage: Supplementary Fig. S16A) and visceral fat (absolute e(g)WAT: Figure 6B, body-weight percentage: Supplementary Fig. S16B) in *Tspan7*-knockout mice were analyzed across age groups—young (6–10 weeks), early middle (20–28 weeks), late-middle (32–35 weeks), and old (49–52 weeks)—and during HF feeding—short-term (2 weeks), middle-term (15–19 weeks), and long-term (27–28 weeks)—separated by sex, as shown in the upper panels. The results of simple linear regression analyses of age-related and HF-induced increases in fat mass are presented in the lower panels (equations for each line are provided in Supplementary Table S3). Data for *Tspan7*-overexpressing mice are shown in Figure 14 (equations for each line are provided in Supplementary Table S3).

**Figure 6:**
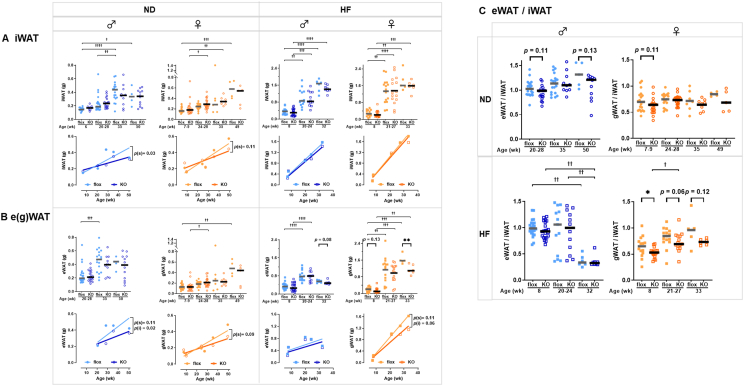
***Tspan7* may be involved in the regulation of fat-storage sites.** (A) iWAT weight, (B) e(g)WAT weight, and (C) e(g)WAT/iWAT ratio in male (blue) and female (orange) *Tspan7*-KO mice (open symbols) and littermate controls (flox, filled symbols) fed an ND (circles) or HF (squares). In the upper panels, symbols represent individual data points, and bars indicate the median (*n* = 5–27 per group). Statistical significance between KO and flox groups was assessed using the Mann–Whitney *U*-test (∗, *p* < 0.05; ∗∗, *p* < 0.01). Differences across time points were analyzed using the Kruskal–Wallis test (^†^, *p* < 0.05; ^††^, *p* < 0.01; ^†††^, *p* < 0.001; ^††††^, *p* < 0.0001). The lower panels display the results of simple linear regression analyses based on individual data from the upper panels. Regression lines for the flox (thinner line) and KO (thicker line) groups are shown. Statistical significance of the slope and intercept differences between the flox and KO regression lines is indicated by *p* values (*p*(s) and *p*(i), respectively). KO, knockout; ND, normal diet; HF, high-fat diet; iWAT, inguinal white adipose tissue; e(g)WAT, epididymal (or gonadal) white adipose tissue. (For interpretation of the references to colour in this figure legend, the reader is referred to the Web version of this article.)

No significant differences in iWAT weight were observed between *Tspan7*-knockout and control mice in either males or females at any age or during any HF feeding period, whether in absolute amounts (Figure 6A upper panel) or as a percentage relative to body weight (Supplementary Fig. S16A upper panel). However, the rate of iWAT increase with aging (slope of the simple linear regression) was significantly lower in *Tspan7*-knockout mice than in that in control mice in males (0.0071 vs. 0.0037 g/week, *p* < 0.05, Figure 6A) and showed a trend toward being lower in females (0.0087 vs. 0.0051 g/week, *p* = 0.11, Figure 6A and Supplementary Table S3). In contrast, no significant differences were observed in the rate of increase due to HF feeding between *Tspan7*-knockout and control mice in either sex (0.048 vs. 0.044 g/week for males, 0.062 vs. 0.059 g/week for females, Figure 6A and Supplementary Table S3). The results, expressed as body weight percentages (Supplementary Fig. S16), were similar to those obtained using absolute weight (Figure 6).

The absolute amount of eWAT tended to be lower in *Tspan7*-knockout mice than that in control mice at late-middle and old ages, as well as during all HF feeding periods (short-term, mid-term, and long-term). Notably, after long-term HF feeding, the absolute amount of eWAT was considerably lower in knockout mice, both in males (*p* = 0.08) and females (*p* < 0.01) (Figure 6B upper panels). The increase in e(g)WAT weight with aging (0.0099 vs. 0.0051 g/week for males, 0.0076 vs. 0.0044 g/week for females, Figure 6B lower panels and Supplementary Table S3) and HF feeding (0.016 vs. 0.014 g/week for males, 0.057 vs. 0.043 g/week for females, Figure 6B lower panels and Supplementary Table S3) was more gradual in *Tspan7*-knockout mice, suggesting suppression of e(g)WAT accumulation in these mice.

In *Tspan7*-overexpression mice, no clear trends were observed in iWAT (Supplementary Fig. S17A). However, eWAT in male *Tspan7*-overexpressing mice exhibited a significantly greater increase after long-term HF feeding than that in control mice (*p* < 0.05, 0.0019 vs 0.011 g/week, Supplementary Fig. S17B lower panels and Supplementary Table S3). This suggests that eWAT accumulation may be accelerated in *Tspan7*-overexpressing mice, although this effect was observed only in males (Supplementary Fig. S17B).

These results indicate that TSPAN7 influences changes in iWAT and eWAT associated with metabolic fluctuations, particularly in eWAT, and may play a role in fat distribution. To further investigate this, we analyzed the visceral-to-subcutaneous fat ratio (e(g)WAT/iWAT ratio), a commonly used indicator for evaluating metabolic disorders and health risks. The e(g)WAT/iWAT ratio in *Tspan7*-knockout mice tended to be lower than that in control mice, regardless of sex, age, or the duration of HF consumption (Figure 6C). *Tspan7* overexpression in males following short-term HF feeding resulted in a significantly higher eWAT/iWAT ratio (*p* < 0.05, Supplementary Fig. S17C).

### 3.6 TSPAN7 regulates fat distribution and its association with metabolic changes

To assess the broader relevance of TSPAN7 in fat distribution, we analyzed the e(g)WAT/iWAT ratio under various experimental conditions. First, we examined *Tspan7*-knockout mice generated by targeting exon 2, whereas all experiments described above (Figure 6 and earlier) involved mice generated by targeting exon 5. Notably, the e(g)WAT/iWAT ratio tended to be lower in both males and females in the exon 2-targeted knockout mice (Figure 7A–C), mirroring the findings in the exon 5-targeted knockout mice (Figure 6C). This trend was particularly pronounced in aged mice of both sexes (*p* < 0.01, Figure 7C). In exon 5-targeted knockout mice, transcripts containing exons 1–4 were still detected, raising the possibility that a truncated TSPAN7 protein with residual function might be expressed. To confirm that the observed phenotype—particularly the altered fat distribution—was due to the loss of TSPAN7, we additionally analyzed exon 2-targeted knockout mice, wherein a more complete loss of TSPAN7 was expected. The consistency of the visceral-to-subcutaneous fat ratio phenotype in both the models strongly supports the role of TSPAN7 in adipose tissue remodeling. Next, we analyzed naturally occurring variations in *Tspan7* expression and body weight across different mouse models. The B6J strain, which exhibits higher *Tspan7* expression (Figure 1B) and gradual weight gain [29], showed a significantly higher eWAT/iWAT ratio than the B6N strain (*p* < 0.05, Figure 7D). Similarly, germ-free mice, which naturally express higher levels of *Tspan7* (Figure 1C) and exhibit a slower rate of weight gain, had a significantly higher eWAT/iWAT ratio than microbiota-colonized mice (*p* < 0.01, Figure 7D). Thus, there appears to be a relationship among the weight-gain phenotype, *Tspan7* expression, and visceral-to-subcutaneous fat ratio. These findings collectively suggest that TSPAN7 influences fat distribution, rather than the absolute amount of fat tissue, with its effects observed in both genetically modified models and natural variations in *Tspan7* expression.

**Figure 7:**
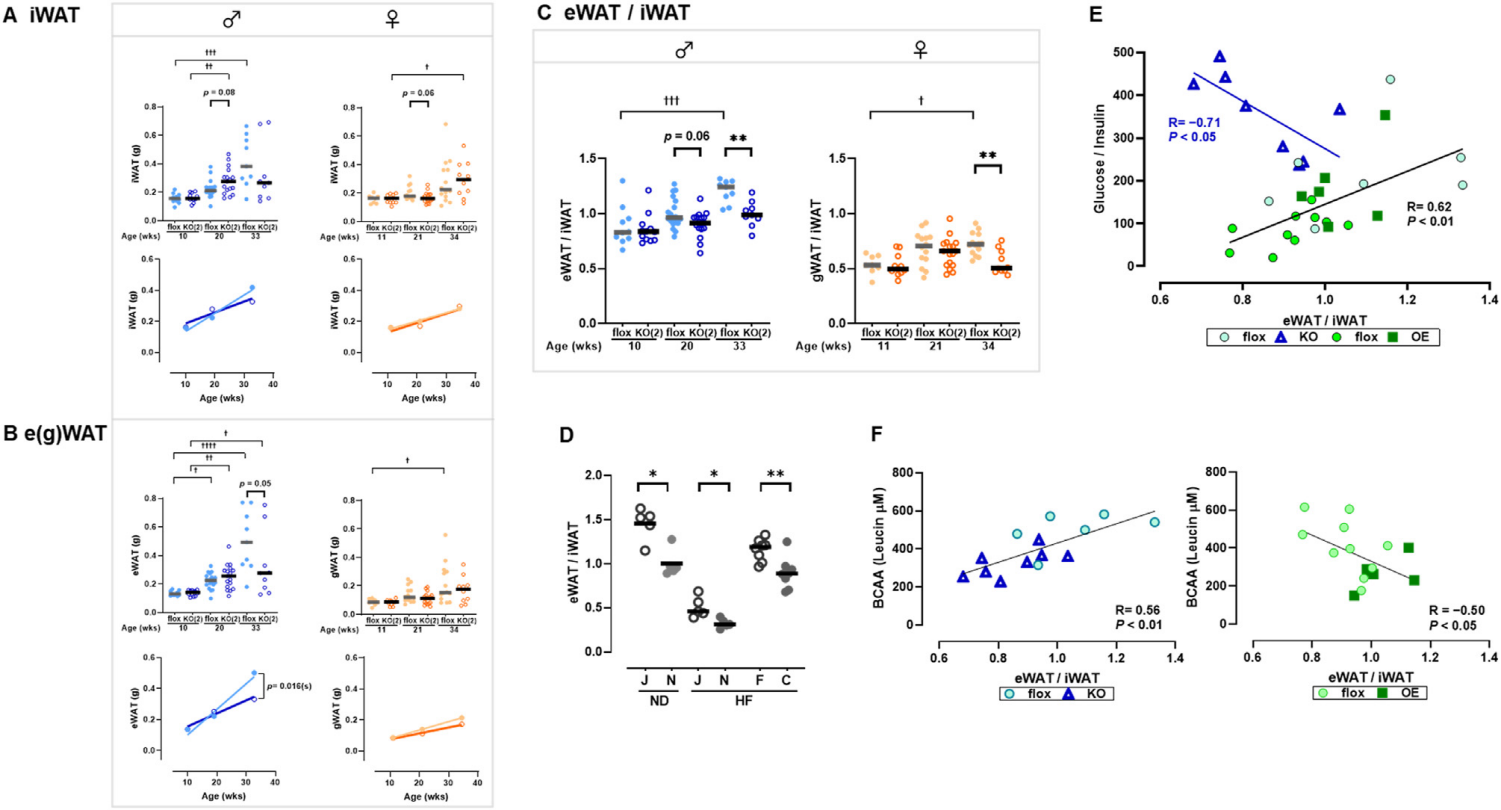
***Tspan7* may influence the distribution of fat accumulation.** (A) iWAT weight, (B) e(g)WAT weight, and (C) e(g)WAT/iWAT ratio in male (blue) and female (orange) ND-fed *Tspan7*-KO mice targeting exon 2 (KO(2), open symbols) and littermate controls (flox, filled symbols). In the upper panels, symbols represent individual data points, and bars indicate the median (*n* = 9–16 per group). Statistical significance between the KO(2) and flox groups was assessed using the Mann–Whitney *U*-test (∗∗, *p* < 0.01). Differences across time points were analyzed using the Kruskal–Wallis test (^†^, *p* < 0.05; ^††^, *p* < 0.01; ^†††^, *p* < 0.001; ^††††^, *p* < 0.0001). The lower panels display the results of simple linear regression analyses based on individual data from the upper panels. The regression lines for the flox (thinner line) and KO(2) (thicker line) groups are shown. Statistical significance of differences in the slope for the flox and KO regression lines is indicated by *p* values (*p*(s)). (D) Comparison of the eWAT/iWAT ratio between C57BL6/J (J) and C57BL6/N (N) mice fed an ND and HF, and between germ-free (F) and germ-colonized (C) mice fed HF. Statistical significance between groups was assessed using the Mann–Whitney *U*-test (∗, *p* < 0.05; ∗∗, *p* < 0.01). (E) Correlation between the eWAT/iWAT ratio and the glucose:insulin ratio and (F) between the eWAT/iWAT ratio and the plasma BCAA concentration (as leucine; μM). Symbols represent individual data points. *Tspan7* KO (blue triangle) and littermate controls (flox, blue circle). *Tspan7* OE mice (green square) and littermate controls (flox, green circle). KO, knockout; OE, overexpressing; ND, normal diet; HF, high-fat diet; iWAT, inguinal white adipose tissue; e(g)WAT, epididymal (or gonadal) white adipose tissue; BCAA, branched-chain amino acid. (For interpretation of the references to colour in this figure legend, the reader is referred to the Web version of this article.)

Building on these findings, we next examined the physiological relevance of the e(g)WAT/iWAT ratio by exploring its relationship with two metabolic parameters: insulin sensitivity (Figure 5M) and BCAA metabolism. The latter is influenced by *Bckdhb*, a gene identified in our study as significantly regulated by *Tspan7* modification (Figure 4B,C). These analyses were performed using data from 8-week-old male *Tspan7*-knockout and -overexpressing mice subjected to short-term HF feeding. Figure 7E presents the correlation between the glucose:insulin ratio, an indicator of insulin sensitivity, and the eWAT/iWAT ratio. In control mice, a positive correlation was observed (*R* = 0.62, *p* < 0.01). However, in knockout mice, which exhibited a lower eWAT/iWAT ratio (Figure 6C), this correlation shifted to a negative relationship (*R* = −0.71, *p* < 0.05). In *Tspan7*-overexpressing mice, which displayed a higher eWAT/iWAT ratio (Supplementary Fig. S17C), the correlation with the glucose:insulin ratio showed a positive trend (*R* = 0.44, *p* = 0.38). Figure 7F illustrates the correlation between blood BCAA concentration and the eWAT/iWAT ratio. Significant correlations were observed in both knockout (*R* = 0.56, *p* < 0.01) and overexpression (*R* = −0.50, *p* < 0.05) mice, highlighting the influence of TSPAN7 on BCAA metabolism. Collectively, these findings indicate that TSPAN7 plays a role in regulating the balance between visceral (e or gWAT) and subcutaneous (iWAT) fat. This balance may have physiological implications for insulin sensitivity and BCAA metabolism, underscoring the role of *Tspan7* in metabolic regulation.

## 4 Discussion

We previously demonstrated that TSPAN7 is involved in body weight regulation [16], yet the underlying mechanisms remained unclear. In this study, we sought to clarify the role of TSPAN7 in metabolic regulation using *Tspan7*-modified mice and genetically altered human cells. Our findings indicate that TSPAN7 contributes to lipid metabolism, thereby playing a role in body weight regulation.

Notably, the loss of TSPAN7 led to a significant shift in the ratio of visceral-to-subcutaneous fat, which was consistently observed across different knockout models and experimental conditions, including both exon 2- and exon 5-targeted mice, as well as variations in naturally occurring TSPAN7 expression, suggesting a role for TSPAN7 adipose tissue remodeling. This altered fat distribution was further associated with two metabolic parameters: insulin sensitivity and BCAA metabolism. In *Tspan7*-knockout mice, the glucose:insulin ratio—an indicator of insulin sensitivity—was negatively correlated with the visceral-to-subcutaneous fat ratio, suggesting a potential physiological association between improved fat distribution and enhanced insulin sensitivity. Moreover, *Tspan7*-knockout mice exhibited reduced blood BCAA levels along with increased expression of *Bckdhb*, an enzyme involved in BCAA catabolism [[36], [37], [38]]. These findings are consistent with previous reports showing that elevated BCAA levels are positively correlated with insulin resistance and visceral fat accumulation [[39], [40], [41], [42]]. Collectively, our results suggest that TSPAN7 modulates BCAA metabolism and may improve insulin responsiveness through the regulation of fat distribution.

In light of these findings, we further investigated the dynamic regulation of TSPAN7 expression in adipose tissue under various metabolic conditions. Specifically, we observed that *Tspan7* expression decreased in response to weight gain induced by aging or HF intake, and that mice with less weight gain exhibited higher *Tspan7* expression compared to those with greater weight gain. These observations not only support the role of TSPAN7 in lipid metabolism and body weight regulation, but also suggest that TSPAN7 may function as a “metabolic switch,” adjusting its expression in response to metabolic states such as energy surplus or deficit, thereby modulating adipose tissue remodeling.

For example, in the present study, young *Tspan7*-knockout mice (6 weeks old) displayed greater fat mass in both subcutaneous and visceral depots compared to control mice under ND conditions. However, under conditions of energy surplus—such as HF feeding or age-related fat accumulation—the rate of fat mass gain was lower in knockout mice than in controls. Furthermore, changes in insulin sensitivity and *Bckdhb* expression due to *Tspan7* modification were also observed only under metabolic stress conditions, including aging and HF intake. Our findings support the hypothesis that TSPAN7 functions as a context-dependent regulator, with its role varying according to metabolic status.

In our previous study [16], we reported that based on body weight measurements from very young mice (3–4 weeks old), those with low *Tspan7* expression tended to become “obese,” whereas those with high expression were more likely to remain “lean.” This may reflect a transient effect of TSPAN7 that manifests before the onset of energy surplus.

Lipid metabolism is a process that dynamically switches between lipolysis and lipid synthesis in response to energy demand and supply, and TSPAN7 may play a role in this switching. Under conditions of energy surplus, TSPAN7 expression is downregulated, which promotes the accumulation of subcutaneous fat while suppressing the accumulation of visceral fat. Although this state may appear as “obesity,” insulin sensitivity is maintained, and it may represent the so-called “metabolically healthy obesity (MHO)” phenotype [43,44]. In contrast, a phenotype with high TSPAN7 expression and a high proportion of visceral fat may be associated with a lower body weight but progressive insulin resistance, leading to a “thin but metabolically unhealthy” (metabolically obese normal weight, MONW)” state [24].

Although TSPAN7 expression was similarly altered in both knockout and overexpressing mice—decreased in the former and increased in the latter—the resulting metabolic phenotypes were not simple opposites of each other. This suggests that the relationship between TSPAN7 expression levels and metabolic outcomes is not linear. Rather than acting as a dose-dependent activator or inhibitor, TSPAN7 may function as a context-dependent regulatory switch whose role is modulated by the metabolic environment. Indeed, while *TSPAN7* knockdown in cultured adipocyte precursors led to notable upregulation of adipogenesis- and lipid droplet-related genes, such changes were not observed in the adipose tissue of knockout or overexpressing mice. This discrepancy may be due to the presence of multiple cell types in adipose tissue—including adipocyte precursors, mature adipocytes, and immune cells—as well as compensatory mechanisms that preserve tissue homeostasis *in vivo*. Collectively, these observations underscore the complexity of TSPAN7’s role in adipose plasticity and suggest that its regulatory impact may be modulated or masked by broader cellular interactions and systemic feedback loops. To elucidate the specific pathways mediating these compensatory responses and their physiological relevance to metabolic homeostasis, further cellular-level investigations are warranted.

Of particular note is that, although *Tspan7* expression was manipulated specifically in adipose tissue, its metabolic effects were observed in other organs as well. While *Tspan7* expression remained unchanged in the liver and skeletal muscle, *Bckdhb* expression in these tissues showed similar changes to those observed in adipose tissue. This suggests that TSPAN7 modification in adipose tissue may exert systemic effects, potentially influencing metabolic regulation in distant organs. Rather than functioning solely as a local regulator in fat, TSPAN7 may act as part of a broader metabolic network that maintains whole-body homeostasis. In particular, elucidating the molecular mechanisms that link enhanced BCAA metabolism (as evidenced by increased *Bckdhb* expression and decreased circulating BCAA levels) with improved fat distribution (reflected by a reduced visceral-to-subcutaneous fat ratio) represents an important avenue for future investigation.

In addition to these systemic observations, our cellular-level analyses provide further insights into the mechanisms by which TSPAN7 may influence lipid metabolism. *Tspan7* expression was relatively high in mitochondria-rich tissues, including the brain, heart, muscle, and kidney. Consistently, our immunofluorescence analysis confirmed the localization of TSPAN7 to mitochondria in hADSCs. These findings suggest that, beyond its established function as a cell membrane-associated protein [45], TSPAN7 may also play roles in mitochondrial functions relevant to lipid metabolism and body weight regulation. Supporting this notion, RNA-seq analysis of *TSPAN7*-knockdown hADSCs revealed altered expression of multiple genes involved in mitochondrial function and energy metabolism. Although we did not directly assess oxygen consumption or mitochondrial activity in this study, these transcriptomic changes imply that TSPAN7 may influence mitochondrial metabolic processes. Further functional validation is necessary to determine whether TSPAN7 directly modulates mitochondrial respiration or interacts with key regulators of oxidative metabolism.

To further investigate the downstream consequences of TSPAN7 modulation, imaging analyses revealed an increased proportion of small adipocytes in *Tspan7*-knockout mice, a trend also observed in adipocytes differentiated from *TSPAN7*-knockdown hADSCs. Proteomic and gene expression analyses identified several lipid droplet- and lipolysis-associated molecules, including PLIN1, PLIN4, LIPE, ABHD5, PNPLA2, and FAF2, as potential mediators of TSPAN7 function. Lipid droplets and mitochondria are both membrane-bound organelles. They interact closely through contact sites that facilitate fatty acid transfer [46,47]. This suggests that TSPAN7 may contribute to lipid metabolism by regulating lipid droplet formation and stabilization. Moreover, *TSPAN7*-knockdown hADSCs exhibited decreased expression of cytoskeletal genes, such as actin and integrins (Supplementary Fig. S18), suggesting that TSPAN7 may also influence lipid droplet dynamics through structural modulation of the cytoskeleton, as it has been reported to interact with cytoskeletal components [48,49]. These findings highlight a potential link between TSPAN7 and the structural and functional remodeling of adipocytes, warranting further studies to explore how these changes impact overall metabolic health.

The metabolic consequences of altered adipocyte morphology, particularly increased proportions of small adipocytes, remain a subject of considerable interest. In general, small adipocytes exhibit higher lipolytic activity, facilitating on-demand energy supply and conferring metabolic benefits, such as improved insulin sensitivity and reduced inflammation [50,51]. Conversely, the accumulation of large adipocytes, typically observed under HF intake conditions, is associated with insulin resistance and other metabolic impairments [52]. Subcutaneous fat depots have greater capacity for expansion through hyperplasia, enabling adaptation to energy surplus by increasing the number of small adipocytes [53,54], whereas visceral fat depots tend to expand through hypertrophy, accumulating larger adipocytes. Consequently, during excess energy intake, fat is preferentially stored in subcutaneous adipose tissue before being deposited as visceral fat [25,53]. In this study, Tspan7-modified mice exhibited an increased proportion of small adipocytes, accompanied by improved insulin sensitivity. This observation suggests that TSPAN7-associated adipocyte remodeling may influence both fat distribution and systemic metabolic adaptation. Elucidating the mechanisms underlying this remodeling process will be critical for advancing our understanding of TSPAN7’s functional role in metabolic regulation.

Given the therapeutic potential of TSPAN7 in metabolic regulation, developing strategies for its targeted modulation is of significant interest. Although TSPAN7 is expressed in multiple tissues and involved in diverse biological processes, our findings highlight that its adipose tissue-specific regulation can lead to systemic metabolic effects, including improved insulin sensitivity. This suggests that targeting TSPAN7 in adipose tissue may offer therapeutic benefits for metabolic disorders such as obesity and type 2 diabetes. Recent advances in selective drug delivery systems, particularly nanotechnology-based carriers and targeted drug delivery systems developed for anti-obesity therapies [[55], [56], [57], [58]], offer promising avenues to enhance the precision and efficacy of such interventions. These approaches may enable the specific modulation of TSPAN7 in adipose tissue while minimizing off-target effects in other organs. Nonetheless, further studies are necessary to refine these strategies and evaluate their feasibility, safety, therapeutic efficacy, and long-term outcomes. Altogether, this study reveals that adipose tissue-specific modulation of TSPAN7 influences systemic metabolic homeostasis and presents a potential therapeutic strategy for metabolic diseases by targeting adipose tissue function.

## CRediT authorship contribution statement

**Shino Nemoto:** Writing – original draft, Investigation, Funding acquisition, Formal analysis, Data curation, Conceptualization. **Kazuyo Uchida:** Investigation. **Tetsuya Kubota:** Investigation. **Manabu Nakayama:** Resources, Funding acquisition. **Yong-Woon Han:** Writing – original draft, Investigation. **Shigeo Koyasu:** Project administration. **Hiroshi Ohno:** Supervision.

## Funding sources

This research was supported by JSPS KAKENHI Grant Numbers JP19K0574 and JP22K11719, and Platform Project for Supporting Drug Discovery and Life Science Research (Basis for Supporting Innovative Drug Discovery and Life Science Research (BINDS)) from AMED under Grant Number JP21am0101119 (support numbers 1403 and 1682).

## Declaration of competing interest

The authors declare that they have no known competing financial interests or personal relationships that could have appeared to influence the work reported in this paper.

## Figures and Tables

**figs1:**
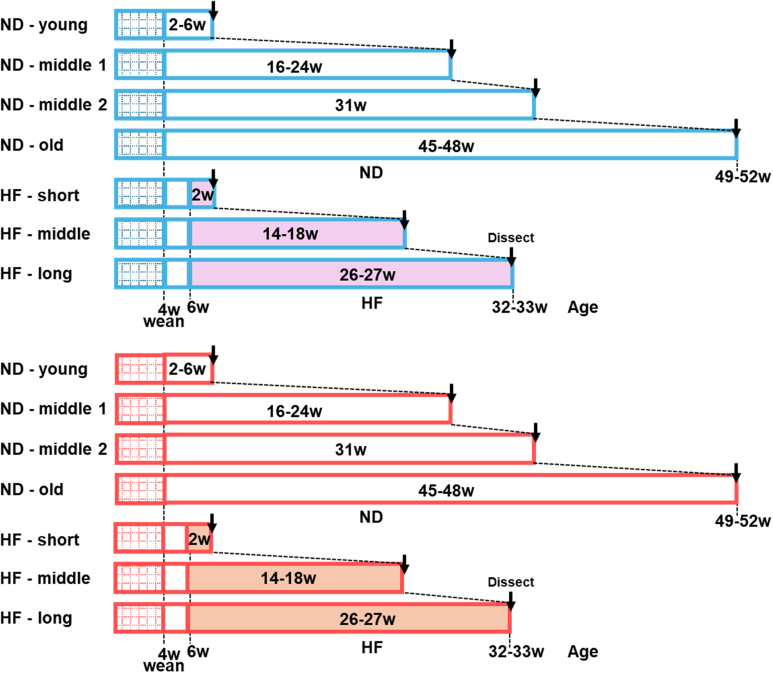


**figs2:**
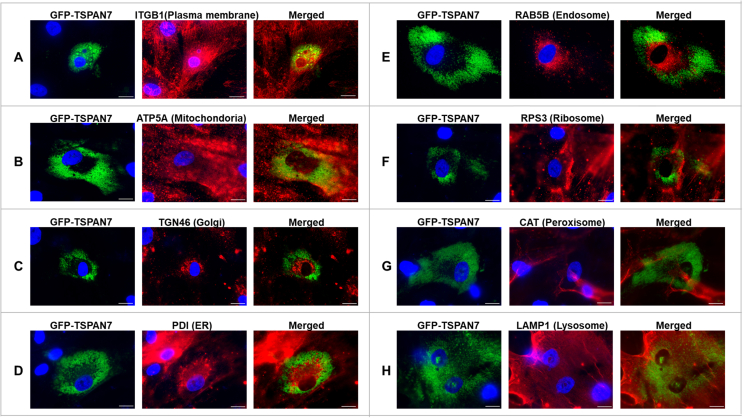


**figs3:**
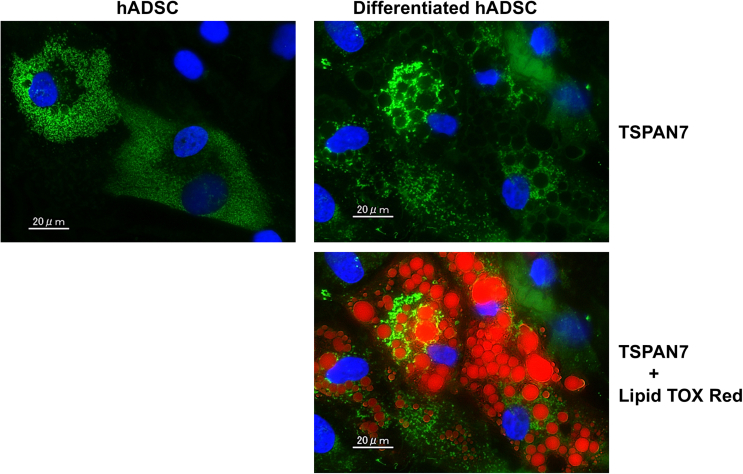


**figs4:**
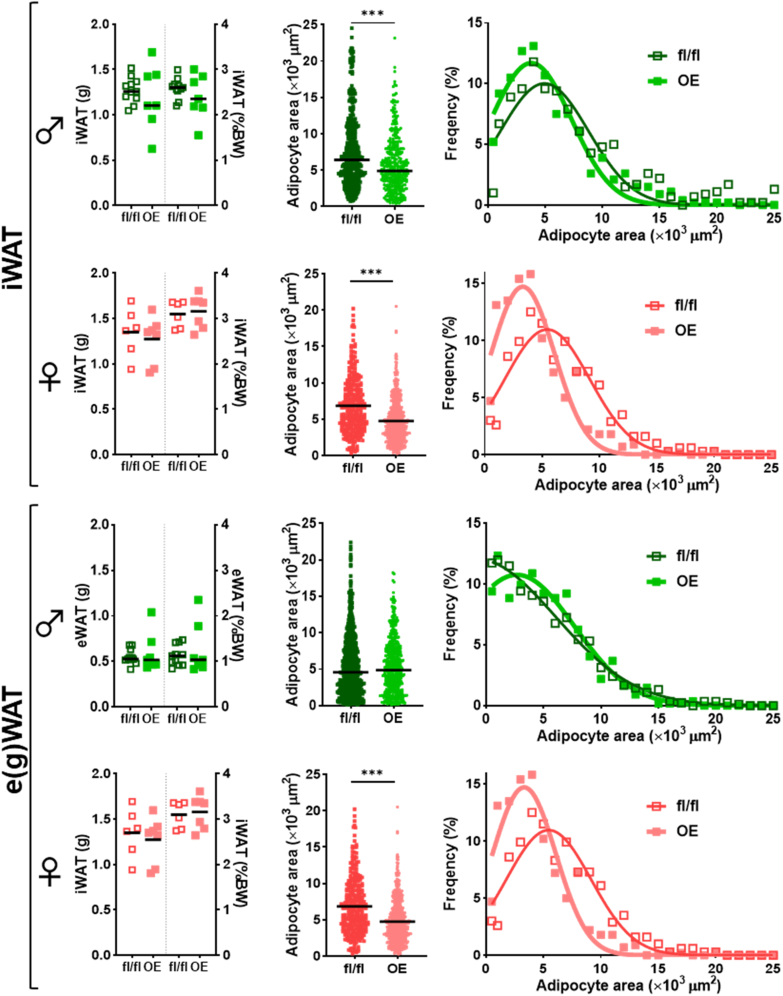


**figs5:**
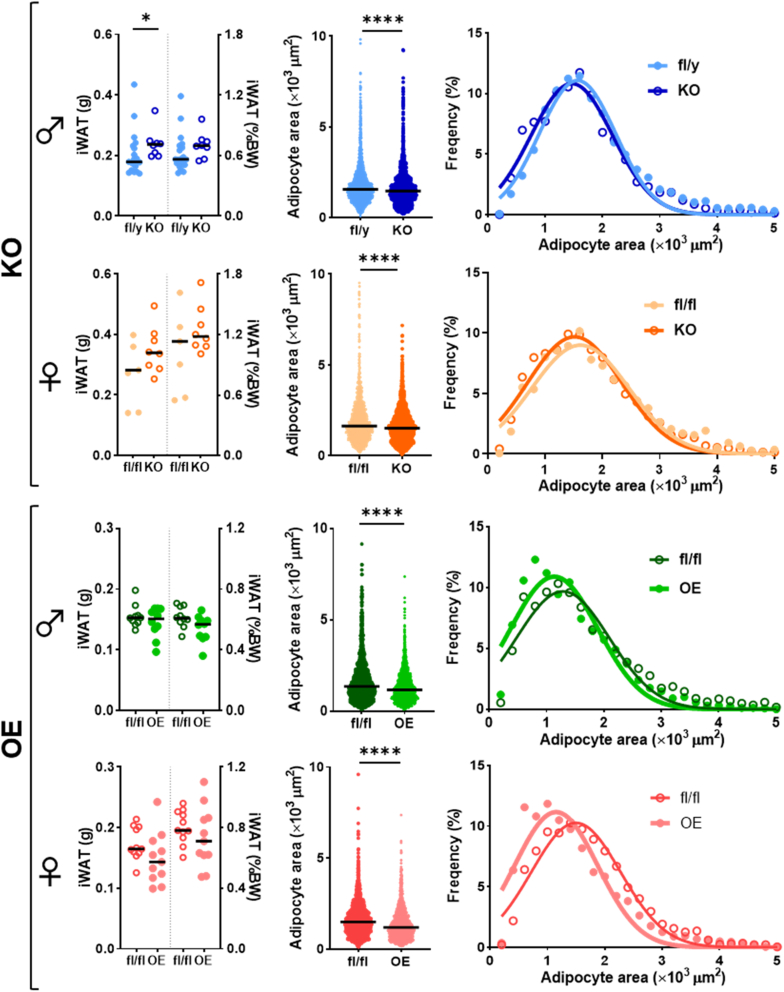


**figs6:**
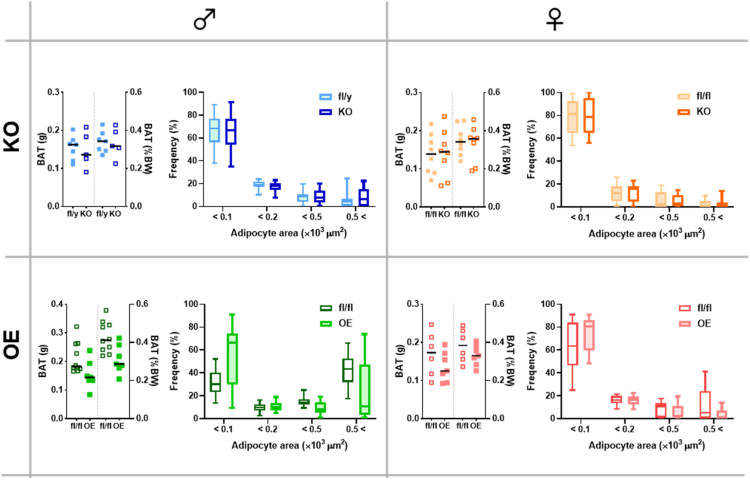


**figs7:**
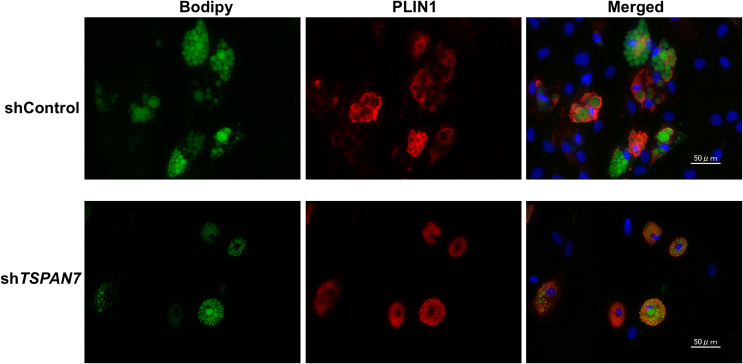


**figs8:**
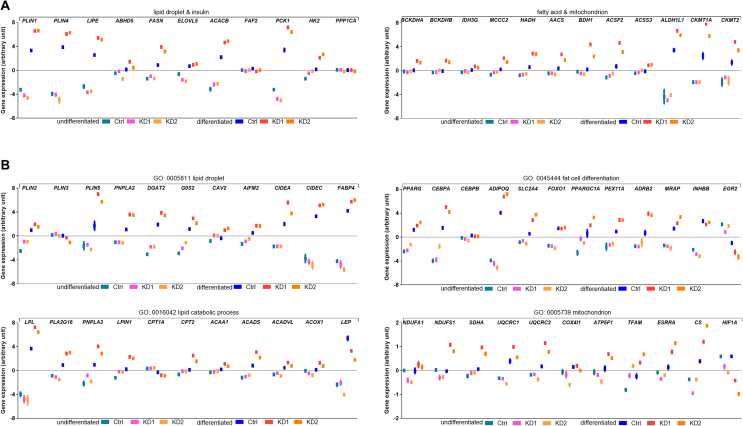


**figs9:**
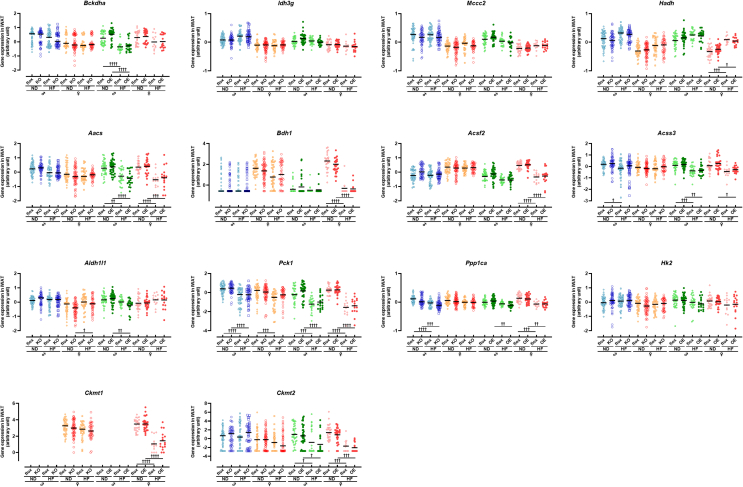


**figs10:**
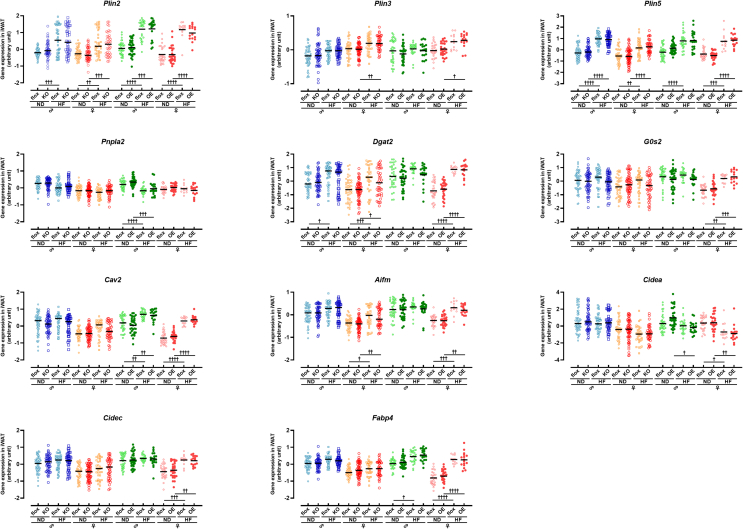


**figs11:**
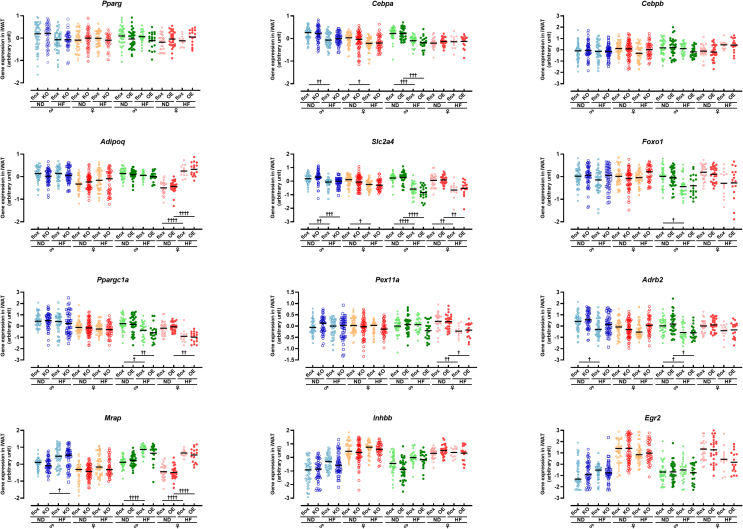


**figs12:**
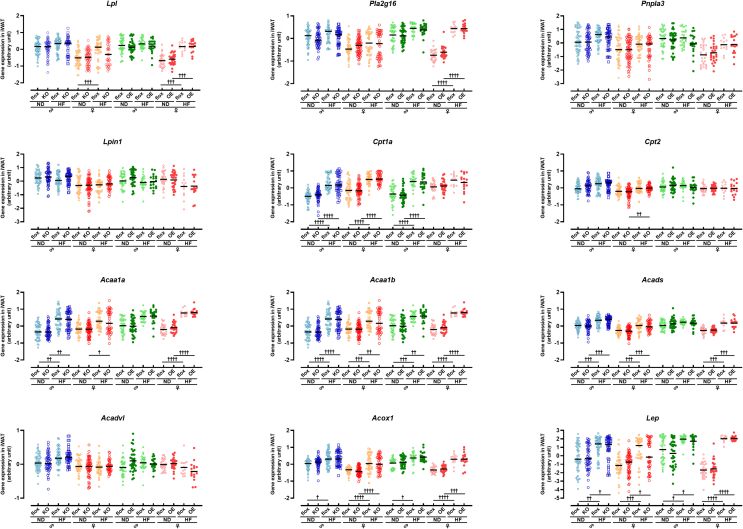


**figs13:**
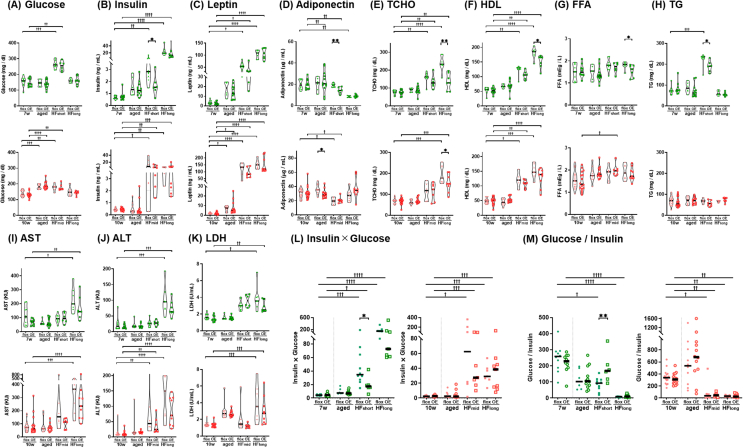


**figs14:**
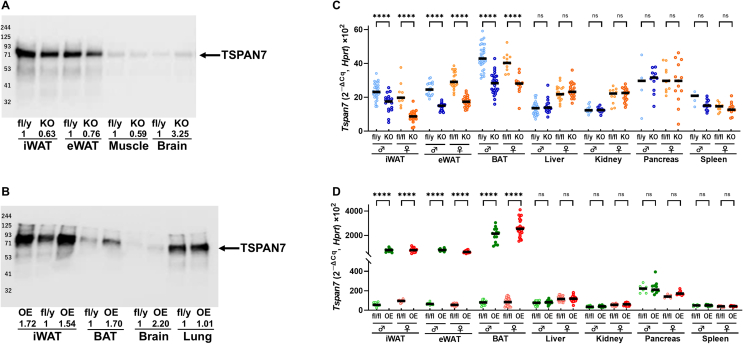


**figs15:**
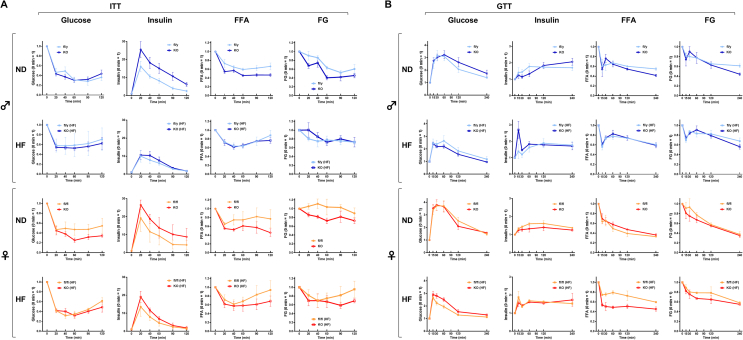


**figs16:**
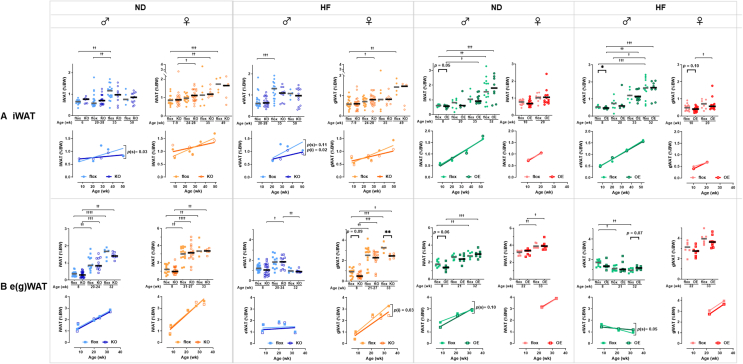


**figs17:**
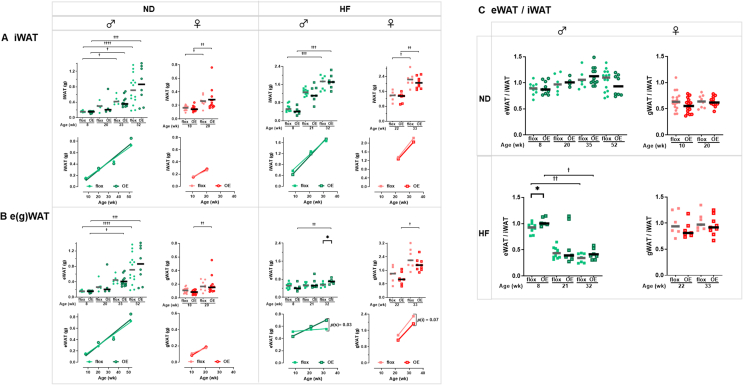


**figs18:**
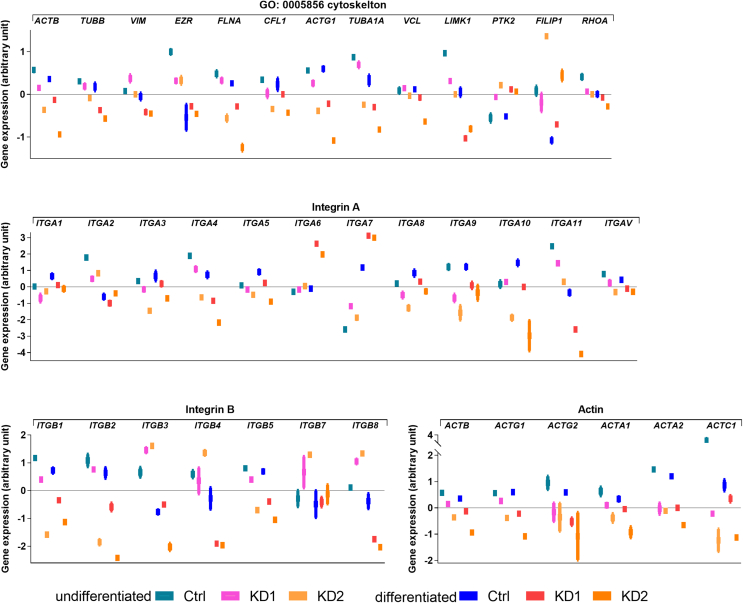


## Data Availability

Data will be made available on request.

## Abbreviations

TSPAN7: tetraspanin7
iWAT: inguinal white adipose tissue
eWAT: epididymal white adipose tissue
gWAT: gonadal white adipose tissue
BAT: brown adipose tissue
HFD: high fat diet
ND: normal diet
TG: triglyceride
FG: free glycerol
FFA: free fatty acid
TCHO: total cholesterol
HDL: high-density lipoprotein cholesterol
AST: aspartate aminotransferase
ALT: alanine aminotransferase
LDH: lactate dehydrogenase
BCAA: branched-chain amino acid
hADSCs: human adipose tissue-derived stem cells
SVF: stromal vascular fraction
PLIN1: perilipin 1
PLIN4: perilipin 4
LIPE: hormone-sensitive lipase
ABHD5: alpha/beta hydrolase domain-containing 5
PNPLA2: patatin-like phospholipase domain containing 2
FAF2: FAS-associated factor 2
BCKDHB: branched-chain α-keto acid dehydrogenase complex subunit B
ACTB: beta actin
GAPDH: glyceraldehyde-3-phosphate dehydrogenase
