# Supplementary material for: Tetraspanin7 in adipose tissue remodeling and its impact on metabolic health
Source: Mol Metab. 2025 May 12;97:102168. doi: 10.1016/j.molmet.2025.102168 (PMC12150174; doi:10.1016/j.molmet.2025.102168)
Supplement: Multimedia component 2 [file mmc2.docx]

Supplementary Figure and Table Captions

**Figure S1** **Experimental timeline.** Seven groups of male (blue) and female (orange) mice were used. At 4 weeks of age, mice were weaned and fed either ND (open boxes) or HF (filled boxes) for different durations. Arrows indicate the time points at which mice were sacrificed, and samples were collected. ND, normal diet; HF, high-fat diet.

**Figure S2** **TSPAN7 localizes to the mitochondria.** Immunofluorescence experiments were performed to confirm subcellular localization of GFP-TSPAN7 relative to that of organelle-specific markers. hADSCs, at 70% confluency, were transduced with GFP-TSPAN7 lentivirus (left panel, green). Two days post-transduction, cells were immunostained with primary antibodies targeting the indicated organelle markers, followed by Alexa Fluor 594-conjugated secondary antibodies (middle panel, red). Nuclei were counterstained with Hoechst　33342 (blue). (A) ITGB1 (plasma membrane marker), (B) ATP5A (mitochondrial marker), (C) TGN46 (Golgi marker), (D) PDI (endoplasmic reticulum marker), (E) RAB5B (early endosome marker), (F) RPS3 (ribosome marker), (G) CAT (peroxisome marker), and (H) LAMP1 (lysosome marker). Scale bar = 20 μm. hADSCs, human adipose-derived stem cells; ITGB1, integrin β1; ATP5A, ATP synthase subunit alpha; TGN46, trans-Golgi network protein 46; PDI, protein disulfide isomerase; RAB5B, Ras-related protein Rab-5B; RPS3, ribosomal protein S3; CAT, catalase; LAMP1, lysosomal-associated membrane protein 1.

**Figure S3** **TSPAN7 localizes around lipid droplets.** hADSCs at 70% confluency were transduced with GFP-TSPAN7 lentivirus (green) and cultured without (left) or with (right) differentiation. Twenty days post-transduction, cells were stained with LipidTOX Red to visualize lipid droplets. Nuclei were counterstained with Hoechst　33342 (blue). hADSCs, human adipose-derived stem cells.

**Figure S4** ***Tspan7* may play a role in lipid droplet dynamics.** Absolute weight and body-weight percentage (left panels, circles indicate individual data points), adipocyte area (middle panels, 500–700 cells counted per group), and frequency distribution of adipocyte area (right panel, Gaussian least-squares fit) in iWAT and e(g)WAT of 21-week-old male (green) and 22-week-old female (pink) *Tspan7*-OE (filled squares) and littermate (fl/fl, open squares) mice. Bars represent the median (*n* = 8–15 per group). Statistical significance was assessed using the Mann–Whitney *U*-test. ***, *p* < 0.001 between fl/fl and OE. iWAT, inguinal white adipose tissue; e(g)WAT, epididymal (or gonadal) white adipose tissue; OE, overexpressing.

**Figure S5** **ND-fed mice also show smaller adipocytes in iWAT.** Absolute weight and body-weight percentage (left panels, circles indicate individual data points), adipocyte area (middle panels, 450–970 cells counted per group), and frequency distribution of adipocyte area (right panel, Gaussian least-squares fit) in inguinal white adipose tissue (iWAT) of 20-week-old male (blue) and 35-week-old female (orange) *Tspan7*-KO (open circles) and littermate (fl/y for males, fl/fl for females, filled circles) mice, 8-week-old male (green) and 10-week-old female (red) *Tspan7*-OE (filled circles) and littermate (fl/fl, open circles) mice fed ND. Bars represent the median (*n* = 6–14 per group). Statistical significance was assessed using the Mann–Whitney *U*-test. ***, *p* < 0.001 between fl/y(fl) and KO (OE). iWAT, inguinal white adipose tissue; ND, normal diet; KO, knockout; OE, overexpressing.

**Figure S6** **BAT does not show any difference in adipocyte size due to *Tspan7* modification.** Adipocytes size in BAT of 20-week-old male (blue) and 21-week-old female (orange) *Tspan7*-KO (open squares and bars) and 21-week-old male (green) and 22-week-old female (red) *Tspan7*-OE (filled squares and bars) mice fed HF. Left panels, BAT weight and body-weight percentage. Squares indicate individual data points. Right panels, frequency distribution of adipocyte area (500–700 cells counted per group). Data are shown as whisker plots, from minimum to maximum with median bars (*n* = 5–7 per group). BAT, brown adipose tissue; KO, knockout; OE, overexpressing; ND, normal diet; HF, high-fat diet.

**Figure S7** **TSPAN7 may be involved in lipid droplet formation.** hADSCs at 70% confluency were transduced with either sh-Control (top panels) or sh-TSPAN7 lentivirus (bottom panels) and cultured for 20 days under differentiation conditions. Lipid droplets were stained with Bodipy (green, left panel). PLIN1 was detected using a primary antibody followed by an Alexa Fluor 594-conjugated secondary antibody (red, middle panel). Nuclei were counterstained with Hoechst　33342 (blue). hADSCs, human adipose-derived stem cells; PLIN1, perilipin 1.

**Figure S8 Gene expression analysis of hADSCs reveals the metabolic role of TSPAN7.** (A) Gene expression levels of differentially expressed proteins upon TSPAN7 knockdown. Proteomics analysis identified terms related to “lipid droplet” and “insulin” (top panel) as well as “fatty acid” and “mitochondrion”. (B) Gene expression levels categorized by gene ontology (GO) terms, including “lipid droplet,” “fat cell differentiation,” “lipid catabolic process”, and “mitochondrion.” hADSCs at 70% confluency were transduced with either sh-Control or sh-TSPAN7 lentivirus and cultured for 20 days. Data are presented for *TSPAN7*-knockdown (red and orange, KD1 and KD2, respectively) and the control (blue, Ctrl). Gene expression was measured using RNA sequencing, and data are shown as whisker plots, representing the range from minimum to maximum (*n* = 2 per group). *PLIN1*, perilipin 1; *PLIN4*, perilipin 4; *LIPE*, hormone-sensitive lipase; *ABHD5*, alpha/beta hydrolase domain-containing 5; *FASN*, fatty acid synthase; *ELOVL5*, ELOVL fatty acid elongase 5; *Acacb*, acetyl-CoA carboxylase beta; *FAF2*, FAS-associated factor 2; *PCK1*, phosphoenolpyruvate carboxykinase 1; *HK2*, hexokinase 2; *PPP1CA*, protein phosphatase 1 catalytic subunit alpha; *BCKDHA*, branched-chain α-keto acid dehydrogenase complex subunit A; *BCKDHB*, branched-chain α-keto acid dehydrogenase complex subunit B; *IDH3G*, isocitrate dehydrogenase (NAD(+)) 3 non-catalytic subunit gamma; *MCCC2*, methylcrotonoyl-CoA carboxylase subunit 2; *HADH*, hydroxyacyl-CoA dehydrogenase; *AACS*, acetoacetyl-CoA synthetase; *BDH1*, 3-hydroxybutyrate dehydrogenase 1; *ACSF2*, acyl-CoA synthetase family member 2; *ACSS3*, acyl-CoA synthetase short-chain family member 3; *ALDHL1*, aldehyde dehydrogenase 1 family member L1; *CKMT1A*, creatine kinase, mitochondrial 1A; *CKMT2*, creatine kinase, mitochondrial 2; *PLIN2*, perilipin 2; *PLIN3*, perilipin 3; *PLIN5*, perilipin 5; *PNPLA2*, patatin-like phospholipase domain containing 2; *DGAT2*, diacylglycerol O-acyltransferase 2; *G0S2*, G0/G1 switch 2; *CAV2*, caveolin 2; *AIFM2*, apoptosis-inducing factor mitochondria-associated 2; *CIDEA*, cell death-inducing DFFA-like effector A; *CIDEC*, cell death-inducing DFFA-like effector C; *FABP4*, fatty acid-binding protein 4; *PPARG*, peroxisome proliferator-activated receptor gamma; *CEBPA*, CCAAT/enhancer-binding protein alpha; *CEBPB*, CCAAT/enhancer-binding protein beta; *ADIPOQ*, adiponectin, C1Q and collagen domain-containing; *SLC2A4*, solute carrier family 2, member 4; *FOXO1*, forkhead box O1; *PPARGC1A*, peroxisome proliferator-activated receptor gamma coactivator 1-alpha; *PEX11A*, peroxisomal biogenesis factor 11 alpha; *ADRB2*, adrenergic receptor beta 2; *MRAP*, melanocortin 2 receptor accessory protein; *INHBB*, inhibin subunit beta B; *EGR2*, early growth response 2; *LPL*, lipoprotein lipase; *PLA2G16*, phospholipase A2 group XVI; *PNPLA3*, patatin-like phospholipase domain containing 3; *LPIN1*, lipin 1; *CPT1A*, carnitine palmitoyltransferase 1A; *CPT2*, carnitine palmitoyltransferase 2; *ACAA1*, acetyl-CoA acyltransferase 1; *ACADS*, acyl-CoA dehydrogenase, short-chain; *ACADVL*, acyl-CoA dehydrogenase, very long chain; *ACOX1*, acyl-CoA oxidase 1; *LEP*, leptin; hADSCs, human adipose-derived stem cells.

**Figure S9 Gene expression analysis of iWAT from** ***Tspan7*-modified mice reveals the metabolic role of TSPAN7.** (A) Expression levels of genes corresponding to differentially expressed proteins identified by proteomics analysis (Figure 3C), including terms related to “lipid droplet,” “insulin,” “fatty acid,” and “mitochondrion,” excluding genes presented in Figure 4. (B–D) Gene expression levels categorized by gene ontology (GO) terms, including (B) “lipid droplet,” (C) “fat cell differentiation,” and (D) “lipid catabolic process.” Data are shown for *Tspan7*-KO male (blue) and female (orange), OE male (green) and female (red), and the respective control mice (light blue, light orange, light green, and pink), fed a normal diet (ND, circles) or high-fat diet (HF, squares). Symbols represent individual data points, and bars indicate the median (*n* = 25–31 per group). Gene expression was measured using RNA sequencing. Statistical significance was determined using the Kruskal–Wallis test. Comparisons between KO (or OE) and flox control groups are not indicated due to lack of significance. Comparisons between ND and HF groups are indicated as ^†^, *p* < 0.05; ^††^, *p* < 0.01; ^†††^, *p* < 0.001; ^††††^, *p* < 0.0001. iWAT, inguinal white adipose tissue; KO, knockout; OE, overexpressing; ND, normal diet; HF, high-fat diet; hADSCs, human adipose-derived stem cells. *Bckdha*, branched-chain α-keto acid dehydrogenase complex subunit A; *Idh3g*, isocitrate dehydrogenase (NAD(+)) 3 non-catalytic subunit gamma; *Mccc2*, methylcrotonoyl-CoA carboxylase subunit 2; *Hadh*, hydroxyacyl-CoA dehydrogenase; *Aacs*, acetoacetyl-CoA synthetase; *Bdh1*, 3-hydroxybutyrate dehydrogenase 1; *Acsf2*, acyl-CoA synthetase family member 2; *Acss3*, acyl-CoA synthetase short-chain family member 3; *Aldhl1*, aldehyde dehydrogenase 1 family member L1; *Pck1*, phosphoenolpyruvate carboxykinase 1; *Ppp1ca*, protein phosphatase 1 catalytic subunit alpha; *Hk2*, hexokinase 2; *Ckmt1*, creatine kinase, mitochondrial 1; *Ckmt2*, creatine kinase, mitochondrial 2; *Plin2*, perilipin 2; *Plin3*, perilipin 3; *Plin5*, perilipin 5; *Pnpla2*, patatin-like phospholipase domain containing 2; *Dgat2*, diacylglycerol O-acyltransferase 2; *G0s2*, G0/G1 switch 2; *Cav2*, caveolin 2; *Aifm2*, apoptosis-inducing factor mitochondria-associated 2; *Cidea*, cell death-inducing DFFA-like effector A; *Cidec*, cell death-inducing DFFA-like effector C; *Fabp4*, fatty acid-binding protein 4; *Pparg*, peroxisome proliferator-activated receptor gamma; *Cebpa*, CCAAT/enhancer-binding protein alpha; *Cebpb*, CCAAT/enhancer-binding protein beta; *Adipoq*, adiponectin, C1Q and collagen domain-containing; *Slc2a4*, solute carrier family 2, member 4; *Foxo1*, forkhead box O1; *Ppargc1a*, peroxisome proliferator-activated receptor gamma coactivator 1-alpha; *Pex11a*, peroxisomal biogenesis factor 11 alpha; *Aadrb2*, adrenergic receptor beta 2; *Mrap*, melanocortin 2 receptor accessory protein; *Inhbb*, inhibin subunit beta B; *Egr2*, early growth response 2; *Lpl*, lipoprotein lipase; *Pla2g16*, phospholipase A2 group XVI; *Pnpla3*, patatin-like phospholipase domain containing 3; *Lpin1*, lipin 1; *Cpt1a*, carnitine palmitoyltransferase 1A; *Cpt2*, carnitine palmitoyltransferase 2; *Acaa1*, acetyl-CoA acyltransferase 1; *Acads*, acyl-CoA dehydrogenase, short-chain; *Acadvl*, acyl-CoA dehydrogenase, very long chain; *Acox1*, acyl-CoA oxidase 1; *Lep*, leptin.

**Figure S10** ***Tspan7* may influence insulin-related metabolic processes.** Metabolic parameters in the plasma of male (green) and female (red) *Tspan7*-OE mice (circles with a thick border) and littermate controls (flox, circles with solid fill) fed an ND (baseline, 6–8 weeks; aged, 34–38 weeks) or HF for different periods (short, 2 weeks; long, 22–30 weeks). (A) Glucose, (B) insulin, (C) leptin, (D) adiponectin, (E) TCHO, (F) HDL, (G) FFA, (H) TG, (I) AST, (J) ALT, (K) LDH, (L) insulin-glucose product, and (M) glucose:insulin ratio. For each box plot, the central mark indicates the median and the symbols represent individual data points (*n* = 20–28 per group). Statistical significance was assessed using the Mann–Whitney *U*-test. *, *p* < 0.05; **, *p* < 0.01; ***, *p* < 0.001 between flox and OE. ^†^, *p* < 0.05; ^††^, *p* < 0.01; ^†††^, *p* < 0.001; ^††††^, *p* < 0.0001 between baseline and other time points within the same genotype. OE, overexpressing; ND, normal diet; HF, high-fat diet; TCHO, total cholesterol; HDL, high-density lipoprotein cholesterol; FFA, free fatty acids; TG, triglyceride; AST, aspartate aminotransferase; ALT, alanine aminotransferase; LDH, lactate dehydrogenase.

**Figure S11 Protein and gene expressions were specifically altered in adipose tissue of** ***Tspan7***-**modified mice generated using the Adipoq-Cre system.** Tissue lysates (50 μg of protein) were analyzed by western blotting, and band intensities were quantified using ImageJ. (A) TSPAN7 expression in iWAT, eWAT, muscle, and brain from 11-week-old male *Tspan7*-KO (KO) and littermate control (fl/y) mouse fed a normal diet (ND). (B) TSPAN7 expression in iWAT, BAT, brain, and lung from 10-week-old male *Tspan7*-OE (OE) and littermate control (fl/fl) mouse fed an ND. (C) (D) Gene expressions levels of *Tspan7* (relative to *Hprt*) in iWAT, eWAT (or gWAT), BAT, liver, kidney, pancreas, and spleen from 8–20 week-old mice, measured using qPCR. Data are presented for *Tspan7*-KO male (blue) and female (orange), OE male (green) and female (red), and the respective control mice (light blue, light orange, light green, and pink). Mice were fed an ND (circles) or a high-fat diet (HF, squares). Symbols represent individual data points, and bars indicate the median (*n* = 5–27 per group). Statistical significance between KO (or OE) and floxed control groups was determined using the Mann-Whitney *U*-test. ****, *p* < 0.0001; ns, not significant. iWAT, inguinal white adipose tissue; eWAT, epididymal white adipose tissue; BAT, brown adipose tissue; muscle, skeletal soleus muscle; Brain, whole tissue; KO, knockout; OE, overexpressing.

**Figure S12** ***Tspan7*** **deletion enhances the suppressive effect of insulin on lipolysis.** (A) Insulin tolerance tests were conducted on ND-fed male and female *Tspan7*-knockout (KO) and littermate control (fl/y and fl/fl) mice at 9 weeks of age and on 10-day HF-fed male and female mice at 11 weeks of age after a 5 h-fast. Mice were intraperitoneally injected with human insulin (HumulinR 0.75 U/kg body weight). Blood samples were collected at 0 (baseline), 20, 40, 60, 90, and 120 min post-injection. (B) Glucose tolerance test was performed on ND-fed male and female *Tspan7*-knockout (KO) and littermate control (fl/y and fl/fl) mice at 8 weeks of age and on 10-day HF-fed male and female mice at 10 weeks of age after an overnight fast (13 h). Mice were administered D-glucose (3 g/kg body weight) via oral gavage. Blood samples were collected at 0 (baseline), 15, 30, 60, 90, 120, and 240 min post-administration. Plasma glucose, insulin, free fatty acids (FFA), and free glycerol (FG) levels were measured. Data are presented as mean ± SD (*n* = 4–5 per group for males, *n* = 3 per group for females). Blue, male KO; light blue, male littermate; orange, female KO; light orange, female littermate. ND, normal diet; HF, high-fat diet.

**Figure S13** **Relative fat depot weights (% body weight) align with absolute weight data.** (A) iWAT and (B) e(g)WAT weight relative to body weight (%BW) in male (blue) and female (orange) *Tspan7*-KO (open symbols) and littermate controls (flox, filled symbols), and in male (green) and female (red) *Tspan7*-OE (symbols with a thicker border) and littermate controls (flox, symbols with solid fill), fed an ND (circles) or HF (squares). In the upper panels, symbols represent individual data points, and bars indicate the median (*n* = 5–27 per group). Statistical significance between KO (OE) and flox groups was assessed using the Mann–Whitney *U*-test (*, *p* < 0.05; **, *p* < 0.01). Differences across time points were analyzed using the Kruskal–Wallis test (^†^, *p* < 0.05; ^††^, *p* < 0.01; ^†††^, *p* < 0.001; ^††††^, *p* < 0.0001). The lower panels display the results of simple linear regression analyses based on individual data from the upper panels. Regression lines for the flox (thinner line) and KO (OE) (thicker line) groups are shown. Statistical significance of differences in the slope and intercept for flox and KO (OE) regression lines is indicated by *p*-values (*p*(s) and *p*(i), respectively). KO, knockout; OE, overexpressing; ND, normal diet; HF, high-fat diet; iWAT, inguinal white adipose tissue; e(g)WAT, epididymal (or gonadal) white adipose tissue.

**Figure S14** ***Tspan7*** **may be involved in the regulation of fat-storage sites.** (A) iWAT weight, (B) e(g)WAT weight, and (C) e(g)WAT/iWAT ratio in male (green) and female (red) *Tspan7*-OE mice (symbols with a thick border) and littermate controls (flox, symbols with solid fill) fed an ND (circles) or HF (squares). Symbols represent individual data points, and bars indicate the median (*n* = 5–27 per group). Statistical significance between OE and flox groups was assessed using the Mann–Whitney *U*-test (*, *p* < 0.05; **, *p* < 0.01). Differences across time points were analyzed using the Kruskal–Wallis test (^†^, *p* < 0.05; ^††^, *p* < 0.01; ^†††^, *p* < 0.001; ^††††^, *p* < 0.0001). The lower panels of (A) and (B) display the results of simple linear regression analyses based on individual data from the upper panels. Regression lines for the flox (thinner line) and KO (thicker line) groups are shown. The statistical significance of the slope and intercept differences between the flox and OE regression lines is indicated by *p*-values (*p*(s) and *p*(i), respectively). KO, knockout; OE, overexpressing; ND, normal diet; HF, high-fat diet; iWAT, inguinal white adipose tissue; e(g)WAT, epididymal (or gonadal) white adipose tissue.

**Figure S15** **Gene expression analysis of hADSCs reveals the involvement of TSPAN7 in the cytoskeletal organization.** Gene expression levels categorized by gene ontology (GO) terms related to “cytoskeleton” (top panel), integrin subunits (middle panel), and actin isoforms (bottom panel) are shown. hADSCs at 70% confluency, were transduced with either sh-Control or sh-TSPAN7 lentivirus and cultured for 20 days. Data are presented for *TSPAN7*-knockdown (red and orange, KD1 and KD2, respectively) and the control (blue, Ctrl). Gene expression was measured using RNA sequencing, and data are shown as whisker plots, representing the range from minimum to maximum (*n* = 2 per group). *ACTB*, actin beta; *TUBB*, tubulin beta class I; *VIM*, vimentin; *FLNA*, filamin A; *CFL1*, cofilin 1; *ACTG1*, actin gamma 1; *TUBA1A*, tubulin Alpha 1a; *VCL*, vinculin; *LIMK1*, LIM domain kinase 1; *PTK2*, protein tyrosine kinase 2; *FILIP1*, filamin A interacting protein 1; *RHOA*, ras homolog family member A; *ITGA1*, integrin subunit alpha 1; *ITGA2*, integrin subunit alpha 2; *ITGA3*, integrin subunit alpha 3; *ITGA4*, integrin subunit alpha 4; *ITGA5*, integrin subunit alpha 5; *ITGA6*, integrin subunit alpha 6; *ITGA7*, integrin subunit alpha 7; *ITGA8*, integrin subunit alpha 8; *ITGA9*, integrin subunit alpha 9; *ITGA10*, integrin subunit alpha 10; *ITGA11*, integrin subunit alpha 11; *ITGAV*, integrin subunit alpha V; *ITGB1*, integrin subunit beta 1; *ITGB2*, integrin subunit beta 2; *ITGB3*, integrin subunit beta 3; *ITGB4*, integrin subunit beta 4; *ITGB5*, integrin subunit beta 5; *ITGB7*, integrin subunit beta 7; *ITGB8*, integrin subunit beta 8; *ACTG2*, actin gamma 2; *ACTA1*, actin alpha 1, skeletal muscle; *ACTA2*, actin alpha 2, smooth muscle; *ACTC1*, actin alpha cardiac muscle 1.

**Table S1** **Protein–Protein interaction network analysis.** Proteins identified via the protein**–**protein interaction analysis using the STRING database are listed in descending order of node degree, representing the number of interactions associated with each protein and indicating their potential connectivity within the network. Proteins are color-coded based on term descriptions: “lipid droplet” in pink, “fatty acid” in yellow, “mitochondrion” in green, and “insulin signaling” in blue.

**Table S2** **Simple linear regression analyses of age-related and HF diet-induced increase in fat mass.** Equations, *R*-squared values, and *p*-values for Figures 6, S13, and S14. ND, normal diet; HF, high-fat diet; iWAT, inguinal white adipose tissue; e(g)WAT, epididymal (or gonadal) white adipose tissue; KO, *Tspan7*-knockout mice; OE, *Tspan7*-overexpression mice; flox, respective control mice.

**Table S3** **Statistical analysis of insulin tolerance tests (ITT) and glucose tolerance tests (GTT) data in *Tspan7*-knockout and littermate control mice.** Statistical analysis results of (A) ITT and (B) GTT performed in normal diet (ND)- and high-fat diet (HF)-fed male and female *Tspan7*-knockout and littermate control mice. Plasma glucose, insulin, free fatty acids (FFA), and free glycerol (FG) levels were analyzed using repeated measures ANOVA based on a general linear model with the Geisser-Greenhouse correction. Multiple comparisons between group means were conducted using Bonferroni method. F-statistics and corresponding P-values for Genotype, Time, and their interaction (Genotype × Time) are shown. P-values less than 0.1 for “Genotype” and “Genotype × Time” are indicated in bold.
